# Path-level interpretation of Gaussian graphical models using the pair-path subscore

**DOI:** 10.1186/s12859-021-04542-5

**Published:** 2022-01-05

**Authors:** Nathan P. Gill, Raji Balasubramanian, James R. Bain, Michael J. Muehlbauer, William L. Lowe Jr., Denise M. Scholtens

**Affiliations:** 1 Feinberg School of Medicine, Northwestern University, Chicago, IL USA; 2 Department of Biostatistics and Epidemiology, University of Massachusetts - Amherst, Amherst, MA USA; 3 Sarah W. Stedman Nutrition and Metabolism Center, Duke University Medical Center, Durham, NC USA; 4 Duke Molecular Physiology Institute, Durham, NC USA; 5 Duke University School of Medicine, Durham, NC USA

**Keywords:** Network analysis, Graphical models, Graph theory, Metabolomics

## Abstract

**Background:**

Construction of networks from cross-sectional biological data is increasingly common. Many recent methods have been based on Gaussian graphical modeling, and prioritize estimation of conditional pairwise dependencies among nodes in the network. However, challenges remain on how specific paths through the resultant network contribute to overall ‘network-level’ correlations. For biological applications, understanding these relationships is particularly relevant for parsing structural information contained in complex subnetworks.

**Results:**

We propose the pair-path subscore (PPS), a method for interpreting Gaussian graphical models at the level of individual network paths. The scoring is based on the relative importance of such paths in determining the Pearson correlation between their terminal nodes. PPS is validated using human metabolomics data from the Hyperglycemia and adverse pregnancy outcome (HAPO) study, with observations confirming well-documented biological relationships among the metabolites. We also highlight how the PPS can be used in an exploratory fashion to generate new biological hypotheses. Our method is implemented in the R package pps, available at https://github.com/nathan-gill/pps.

**Conclusions:**

The PPS can be used to probe network structure on a finer scale by investigating which paths in a potentially intricate topology contribute most substantially to marginal behavior. Adding PPS to the network analysis toolkit may enable researchers to ask new questions about the relationships among nodes in network data.

**Supplementary Information:**

The online version contains supplementary material available at 10.1186/s12859-021-04542-5.

## Background

Construction of undirected biological networks from continuous cross-sectional data for multiple features (nodes) is dominated by two approaches for determining edge existence and/or edge weights. The first, correlation networks, is based on thresholded pairwise Pearson correlations. Many recent methods, on the other hand, have been based on Gaussian graphical modeling (GGM), which prioritize estimation of conditional pairwise dependencies among nodes in the network. While this latter class of methods is widely used, challenges remain on how specific paths through the resultant network contribute to the overall marginal or ‘network-level’ correlations. For biological applications, understanding these relationships is particularly relevant for parsing structural information contained in complex subnetworks. In this paper, we propose a method for interpreting GGMs at the level of individual network paths based on the relative importance of such paths in determining the Pearson correlation between their terminal nodes. The method is based on a representation of the marginal correlation between two nodes in terms of GGM topology that is developed in the “Methods” section. We will begin by giving some background on both correlation networks and GGMs to motivate this development.

**Fig. 1:**
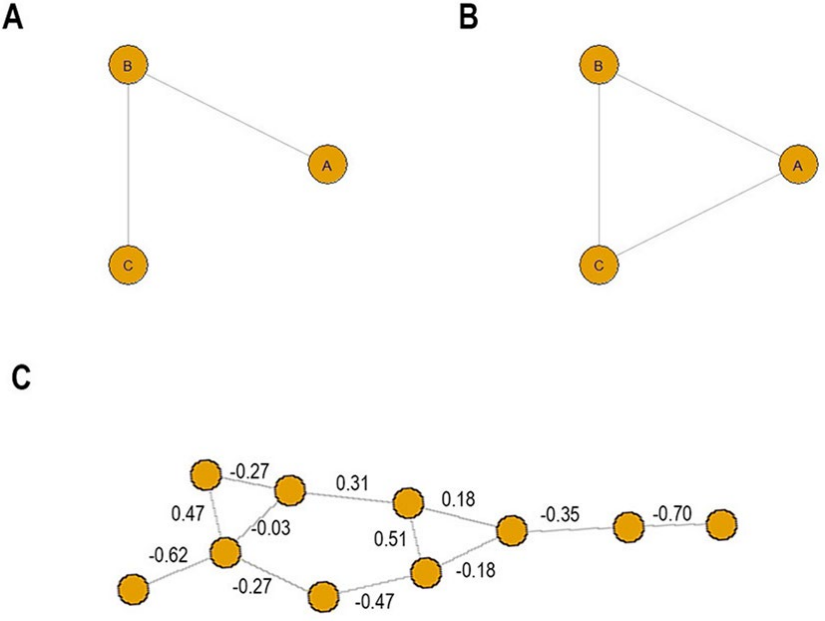
**a** Network of direct interactions/graphical model. **b** Possible correlation network. **c** 10 node network used in simulation study

Correlation networks are typically constructed by first computing the sample Pearson correlation between all pairs of nodes in a dataset, and then drawing an edge between those nodes whose correlation exceeds some threshold [1–7]. This method is easy both to understand and to implement, but it has a major drawback that is best illustrated with an example. Consider three biological compounds A, B, and C, where A upregulates B and B upregulates C. Figure 1a depicts the direct relationships among A, B, and C, but a correlation network might look different. Since A and B are positively correlated, there would be an edge between A and B, and likewise between B and C. However, an edge between A and C may also be observed, as depicted in Fig. 1b–for although A and C do not affect each other directly, they are nevertheless positively correlated through their relationship with B.

Edges like the one between A and C in Fig. 1b that don’t map to a direct biological dependency are a potential drawback to using sample Pearson correlations for network construction. As the example illustrates, the root of the problem is that the correlation between two nodes captures the net action of a complex network. In this sense, the Pearson correlation for a pair of nodes can be viewed as a *network level* statistic [6, 8]. To avoid this issue, many researchers construct networks based instead on the partial correlation between two nodes: the correlation of the residuals for each node in the pair of interest after linear regression of the values of each node on the values of all other nodes, or, equivalently, the correlation of two nodes conditional on all other nodes [2, 8–13]. Such networks are known as Gaussian graphical models (GGMs). Because edges in these networks represent independent relationships conditional on all other network features, they are often posited to represent underlying biological mechanisms [8]. In the example depicted in Fig. 1, because C does not depend on A when B is held constant, the partial correlation between A and C would be zero, and there is no edge between them.

This paper addresses two fundamental challenges in the use of correlation networks and GGMs for the analysis of biological data. The first challenge is the absence of a characterization of the relationship between these two methods that is both precise and biologically meaningful. It is well-established that correlation and partial correlation are related by matrix inversion [14]. If $\mathbf{P } = \{\pi _{ij}\}$ is a partial correlation matrix, where $\pi _{ij}$ denotes the partial correlation between nodes *i* and *j*, then the correlation matrix $\mathbf{C }$ is given by

1 $$\begin{aligned} \mathbf{C } = \mathbf{D }^{-1}\mathbf{A }^{-1}\mathbf{D }^{-1}, \end{aligned}$$

where the $(i,j)$ entry of $\mathbf{A }$ is given by

2 $$\begin{aligned} a_{ij} = {\left\{ \begin{array}{ll} - \pi _{ij} &{} i \ne j \\ 1 &{} i = j \end{array}\right. }, \end{aligned}$$

and $\mathbf{D }$ is a diagonal matrix with $d_{ii}$ equal to the square root of the (*i*, *i*) entry of $\mathbf{A }^{-1}$. (Note that $\mathbf{A }$ is the normalized precision matrix - we have presented it in this way to simplify computations later). Specifically, the correlation matrix can be obtained by inverting the partial correlation matrix with signs of the off-diagonal entries flipped, and normalizing by dividing row $i$ and column $j$ by the square roots of the $i\mathrm{th}$ and $j\mathrm{th}$ diagonal elements (i.e. the same normalization used to pass from covariance to correlation).

The relationship between $\mathbf{P }$ and $\mathbf{C }$ is fully mathematically characterized by (1), but the formula provides little insight for interpreting relationships among individual edges and nodes. In this paper, we demonstrate the ability to express correlation in terms of products of partial correlations that correspond to network topology.

The second challenge this paper addresses is the difficulty of interpreting the results of a GGM analysis. A description of overall network topology (e.g. “scale-free”) is frequently the stopping point, perhaps with some commentary on whether pairs of nodes of interest are connected or not. Depending on the scientific context, this may not be satisfying, especially if there is interest in parsing the structural information in complex subnetworks. With the goal of finer scale, i.e. path-level, interpretation in mind we leverage our characterization of the relationship between GGMs and correlation networks to propose the pair path subscore (PPS), a method of scoring individual network paths in a GGM based on their relative importance in determining the overall network-level correlation between their terminal nodes.

The more interpretable relationship between correlation and partial correlation, as well as PPS, is developed in “Methods” section. The PPS methodology is demonstrated on both simulated data and metabolomics data from the Hyperglycemia and Adverse Pregnancy Outcome (HAPO) Study [15] in “Results” section. Many of the HAPO study metabolites have well-documented biological relationships, and these were used to validate the PPS. The hypothesis-generation potential of PPS is then demonstrated by comparing by comparing networks obtained at different time points during an oral glucose tolerance test as part of the HAPO study, as well as between mothers and newborn babies. Finally, “Discussion and conclusions” section provides a summary and discussion.

## Methods

To motivate the general relationship between correlation and partial correlation, consider a simple three node GGM with partial correlation matrix $\mathbf{P }$, such that,

3 $$\begin{aligned} \mathbf{P } = \begin{pmatrix} 1 &{}\pi _{12} &{} \pi _{13} \\ \pi _{12} &{} 1 &{} \pi _{23} \\ \pi _{13} &{} \pi _{23} &{} 1 \\ \end{pmatrix}. \end{aligned}$$

Application of equation (1) results in correlation matrix **C** defined by

4 $$\begin{aligned} \mathbf{C } = \begin{pmatrix} 1 &{} \frac{\pi _{12}+\pi _{13}\pi _{32}}{\sqrt{1-\pi _{23}^2}\sqrt{1-\pi _{13}^2}} &{} \frac{\pi _{13} + \pi _{31}\pi _{12} }{\sqrt{1-\pi _{21}^2}\sqrt{1-\pi _{13}^2}} \\ \frac{\pi _{12}+\pi _{13}\pi _{32}}{\sqrt{1-\pi _{23}^2}\sqrt{1-\pi _{13}^2}} &{} 1 &{} \frac{\pi _{23} + \pi _{21}\pi _{13}}{\sqrt{1-\pi _{21}^2}\sqrt{1-\pi _{13}^2}} \\ \frac{\pi _{13} + \pi _{31}\pi _{12} }{\sqrt{1-\pi _{21}^2}\sqrt{1-\pi _{13}^2}} &{} \frac{\pi _{23} + \pi _{21}\pi _{13}}{\sqrt{1-\pi _{21}^2}\sqrt{1-\pi _{13}^2}} &{} 1 \end{pmatrix}. \end{aligned}$$

Consider the $(1,2)$ entry of this matrix,

5 $$\begin{aligned} c_{12} = \frac{\pi _{12}+\pi _{13}\pi _{32}}{\sqrt{1-\pi _{23}^2}\sqrt{1-\pi _{13}^2}}. \end{aligned}$$

**Fig. 2:**
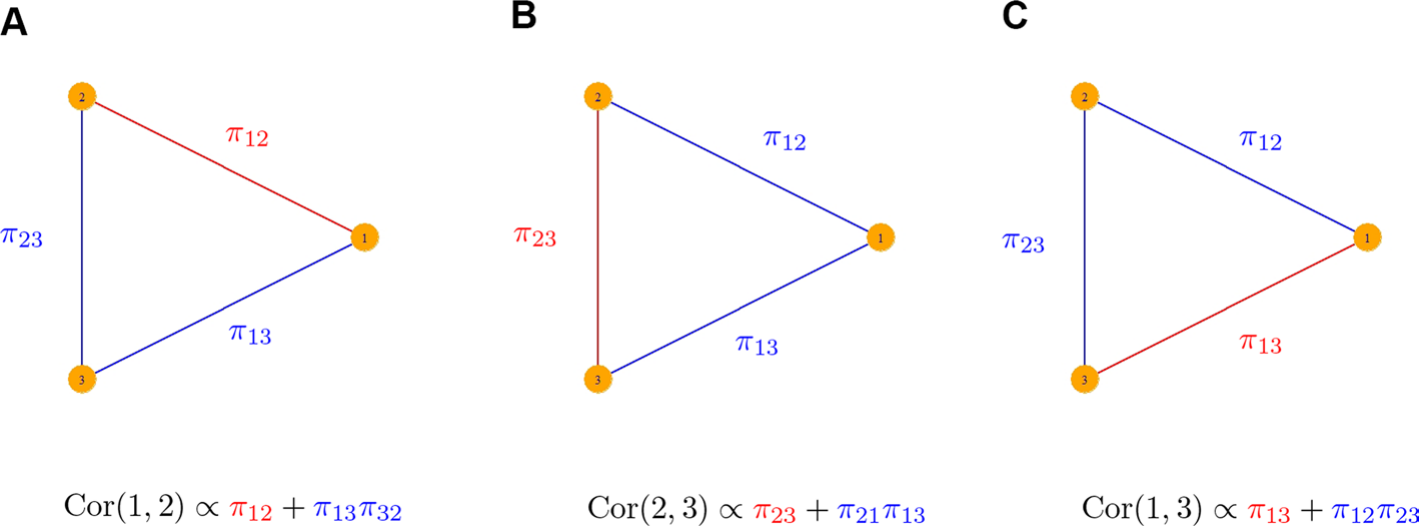
The three panels show the possible pairings of nodes in the simple case of a three node network, with $\pi _{ij}$ denoting the partial correlation between nodes *i* and *j*. In each case, the marginal correlation between the nodes is proportional to the sum of the products of partial correlations along the two network paths connecting them

The numerator has a nice interpretation in terms of the network topology: it is a linear combination of the products of the partial correlations along every path in the network connecting nodes 1 and 2. A “path” is an ordered (but not directed) list of nodes, such that each node in the list is connected by an edge to the previous node in the list, and no edge appears more than once (i.e. there are no loops). The length of a path $p$, denoted $|p|$, is the number of edges in the path. In particular, a single edge is a path of length 1. In the formula above, the first term, $\pi _{12}$, is the product of partials along the length 1 path consisting of the edge between nodes 1 and 2. The second term, $\pi _{13}\pi _{32}$, is the product of the two partials along the path of length 2 from node 1 through node 3 to node 2. The same interpretation holds for the other entries of $\mathbf{C }$ (see Fig. 2).

A similar pattern holds for networks of arbitrary size. Suppose the partial correlation matrix of an $n$-node GGM is

6 $$\begin{aligned} \mathbf{P } = \begin{pmatrix} 1 &{} \pi _{12} &{} \pi _{13} &{} \cdots &{} \pi _{1n} \\ \pi _{12} &{} 1 &{} \pi _{23} &{} \cdots &{} \pi _{2n} \\ \pi _{13} &{} \pi _{23} &{} 1 &{} \cdots &{} \pi _{3n} \\ \vdots &{} \vdots &{} \vdots &{} \ddots &{} \vdots \\ \pi _{1n} &{} \pi _{2n} &{} \pi _{3n} &{} \cdots &{} 1 \end{pmatrix}. \end{aligned}$$

Using the notation (2) from the introduction, let

7 $$\begin{aligned} \mathbf{A } = \begin{pmatrix} 1 &{} -\pi _{12} &{} -\pi _{13} &{} \cdots &{} -\pi _{1n} \\ -\pi _{12} &{} 1 &{} -\pi _{23} &{} \cdots &{} -\pi _{2n} \\ -\pi _{13} &{} -\pi _{23} &{} 1 &{} \cdots &{} -\pi _{3n} \\ \vdots &{} \vdots &{} \vdots &{} \ddots &{} \vdots \\ -\pi _{1n} &{} -\pi _{2n} &{} -\pi _{3n} &{} \cdots &{} 1 \end{pmatrix}. \end{aligned}$$

Let ${\mathscr {P}}_{ij}$ denote the set of all possible paths between nodes $i$ and $j$ in the network. For $p \in {\mathscr {P}}_{ij}$, let $\tau _p \in {\mathbb {R}}$ denote the product of entries of the partial correlation matrix $\mathbf{P }$ along the path $p$, that is,

8 $$\begin{aligned} \tau _p = \Pi _{(k,l) \in p} \pi _{kl}. \end{aligned}$$

The notation $(k,l) \in p$ means that nodes $k$ and $l$ appear sequentially in path $p$. Let $\mathbf{A }_{p^*}$ denote the submatrix of $\mathbf{A }$ with the nodes appearing in path $p$ removed. In particular, $\mathbf{A }_{i^*}$ is the submatrix of $\mathbf{A }$ with only row and column $i$ removed. Note that because $\mathbf{A }_{p^*}$ is a principal submatrix of the positive definite matrix $\mathbf{A }$, it is also positive definite, and hence full rank. Let $|\cdot |$ denote the determinant. Then

9 $$\begin{aligned} \text {Cor}(i, j) = \sum _{p \in {\mathscr {P}}_{ij}}\tau _{p}\frac{|\mathbf{A }_{{p^*}}|}{\sqrt{|\mathbf{A }_{{i^*}}|| \mathbf{A }_{{j^*}}|}}. \end{aligned}$$

A detailed derivation is provided in the appendix.

We emphasize that the novelty here is not in the mathematics itself, which is little more than matrix inversion, but in the algebraic representation of that matrix inversion in terms of the network topology. When expressed this way, the correlation between two nodes has the same intuitive representation seen in the three-node case as a sum of terms each corresponding to a (suitably weighted) product of partial correlations along a particular network path connecting those nodes. In other words, it is a signed and weighted linear combination of products of partial correlations along all paths in the network that connect the two nodes. From a biological point of view, this formulation gives clarity on how correlation measures the combined action of the entire network. It is similar in spirit to Wright’s method of path coefficients [16], which uses directed acyclic graphs to determine the contributions of a set of independent variables to the variance of a dependent variable. This is done via products of regression coefficients along paths in the graph. However, the setting here is an undirected GGM rather than a directed, possibly causal graph, and we are interested in a symmetric decomposition of the correlation between two variables rather than regression.

### The pair-path subscore

We propose using an algebraic score based on (9) relating correlation and partial correlation to probe network structure at the level of individual paths. For a fixed pair of nodes $i$ and $j$, and a path $p \in {\mathscr {P}}_{ij}$, let

10 $$\begin{aligned} \gamma _p = \tau _{p}\frac{|\mathbf{A }_{{p^*}}|}{\sqrt{|\mathbf{A }_{{i^*}}|| \mathbf{A }_{{j^*}}|}}. \end{aligned}$$

Then (9) can be written as

11 $$\begin{aligned} \text {Cor}(i, j) = \sum _{p \in {\mathscr {P}}_{ij}}\gamma _p, \end{aligned}$$

where $\text {Cor}(i,j) \in [-1, 1]$. For any path $p \in {\mathscr {P}}_{ij}$ (the set of all paths with $i$ and $j$ as terminal nodes), we associate a pair-path subscore (PPS) given by

12 $$\begin{aligned} s_p = \frac{|\gamma _p|}{\sum _{p \in {\mathscr {P}}_{ij}}|\gamma _p|}. \end{aligned}$$

**Fig. 3:**
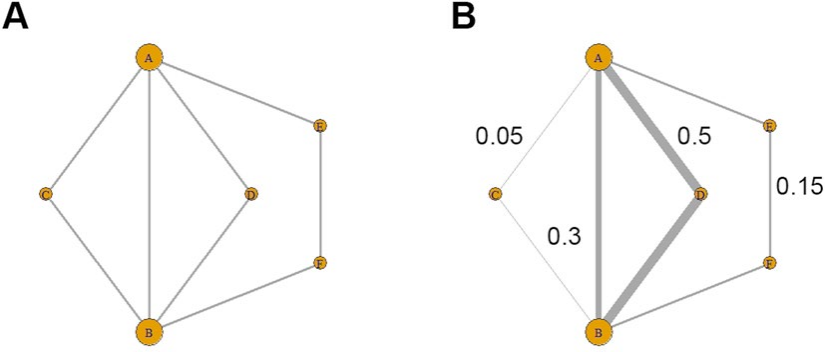
A schematic showing how different paths in a GGM can contribute to the correlation between two nodes **a** and **b**. Panel **a** shows the paths connecting **a** and **b**, while panel **b** shows the same paths with possible PPS values (note that these sum to 1). From PPS, we learn that the edge between **a** and **b**, and the path through node **d**, are the largest contributors to the marginal correlation between nodes **a** and **b**

PPS measures the relative contribution of path $p$ to the correlation between nodes $i$ and $j$. It has the properties that $0 \le s_p \le 1$ and $\sum _{p \in {\mathscr {P}}_{ij}}s_p = 1$. A path with PPS near 1 plays a large role in determining the correlation between its terminal nodes, while a path with PPS near 0 plays a small role. By scoring in this way, we can identify which paths among many in a complicated network play the largest role in determining network level correlation between a given pair of nodes (Fig. 3).

Equation (9) had previously been stated in [17]. This paper goes on to use what we have called $\gamma _p$ (equation (10), the unsigned numerator in PPS) as a measure of the contribution of a path to the correlation between its terminal nodes. Additional papers [18, 19] discuss the interpretation of these path weights and expand the concept to path-level decompositions of other measures of association between network nodes. We note that, in these papers, the quantity of interest is $\gamma _p$, whereas in this paper the quantity of interest is the PPS (12), and we provide a detailed account of its properties and behavior when applied to real data. A key difference between the PPS and the $\gamma _p$ is that the PPS measures the proportion of the correlation attributable to a path, whereas $\gamma _p$ gives the raw contribution. Also distinctive in our paper is the availability of a software package to implement PPS. Our software can also be used to implement the methods of [17–19], since the $\gamma _p$ themselves are also available.

#### PPS and other GGM interpretation techniques

The PPS provides a way to understand which paths between a fixed pair of nodes in a GGM are the most important. In this sense, it is most similar to a technique like weighted shortest path, which minimizes the sum of a positive score along the edges connecting two nodes. Both methods are capable of identifying key paths between a fixed pair of nodes in a GGM, but PPS provides more context, and is directly interpretable as the fraction of the marginal correlation between two nodes attributable to that path. This is because it is based on the precise mathematical relationship (9) between partial and marginal correlation, rather than an edgewise loss function that may not take the entire GGM topology into account.

The modularity coefficient is another GGM interpretation technique, in which clustering based on a particular classification of nodes is assessed. However, this classification must be prespecified, whereas PPS makes no assumptions about which nodes constitute important paths. If a particular grouping of nodes is found to be a cluster based on its modularity coefficient, PPS could be applied to pairs of nodes from that cluster to identify crititcal paths through the cluster. For example, for the acylcarnitine subnetwork of our example HAPO metabolic network, the modularity coefficient is 0.36, indicating some clustering. However, it is the subsequent PPS analysis that allows us to pick out important paths between nodes in the cluster (see “Results” section, specifically Fig. 9). More broadly, any clustering algorithm could be applied prior to PPS analysis. Pairs of nodes in an identified cluster would serve as natural endpoints for PPS analysis and probing of network relationships at the finer scale of individual paths. This approach may be particularly useful for settings in which there is minimal prior structural knowledge about the network.

Note also the difference in using PPS vs the partial correlation to analyze length 1 paths, i.e. edges, between two nodes. The partial correlation is the correlation conditional on the other nodes - it tells us whether or not there is an association between the nodes independently of all the others. What (9) shows, however, is that this conditional relationship is only one component of the total marginal correlation, which is influenced by all the paths in the network connecting the nodes in question. The PPS measures how large this component is relative to the others. For example, two pairs of nodes could both have a partial correlation of 0.1, say, but a PPS for the edge between them of 0.1 and 0.8 respectively. The low edge PPS of the first pair would indicate that the partial correlation is a relatively small component of the total correlation, and many other network paths contribute as well. On the other hand, the large edge PPS of the second would indicate that the direct edge is itself responsible for a large proportion of the association. In fact, we observe behavior like this in our example HAPO metabolic network. Edges connecting acylcarnitines tend to have a low PPS, while those connecting two amino acids tend to have a large PPS (see “Results” section, specifically Fig. 7).

#### Estimation of PPS from empirical data

Generally, the partial correlation matrix $\mathbf{P }$ of a GGM of interest is unknown, and must be estimated from empirical data. Given such an estimate $\hat{\mathbf{P }} = \{{\hat{\pi }}_{ij}\}$ of the partial correlation matrix, we can form estimates ${\hat{\gamma }}_p$ of the $\gamma _p$ in (10) by substituting ${\hat{\pi }}_{ij}$ for $\pi _{ij}$. We can then estimate the PPS $s_p$ of a path $p$ by

13 $$\begin{aligned} {\hat{s}}_p = \frac{|{\hat{\gamma }}_p|}{\sum _{p \in {\mathscr {P}}_{ij}}|{\hat{\gamma }}_p|}. \end{aligned}$$

In the next section, we will demonstrate this estimation procedure on both real and simulated data using two different GGM estimation techniques: inversion of the estimated Pearson correlation matrix (when $n > p$) and graphical lasso [20], a widely-used $L^1$-penalized method for estimating sparse Gaussian graphical models.

#### Computational considerations

For large and/or complex networks, exhaustively computing ${\mathscr {P}}_{ij}$, and consequently $\sum _{p \in {\mathscr {P}}_{ij}} |\gamma _p|$, the denominator in (13), can be very expensive. In order to make computation of (13) feasible, we recommend only computing paths up to a certain length, rather than all possible paths. In other words, we replace (13) with

14 $$\begin{aligned} {\hat{s}}_p^K = {\left\{ \begin{array}{ll} \frac{|{\hat{\gamma }}_p|}{\sum _{p \in {\mathscr {P}}^K_{ij}}|{\hat{\gamma }}_p|} &{} |p| \le K \\ 0 &{} |p| > K \end{array}\right. }, \end{aligned}$$

where *K* is a positive integer chosen by the user, and ${\mathscr {P}}^K_{ij} = \{p \in {\mathscr {P}}_{ij} : |p| \le K\}$. In doing so, we reduce the worst-case (i.e. a fully-connected network) complexity from ${\mathcal {O}}(N!)$ to ${\mathcal {O}}(N!/(N-K)!)$, a considerable reduction. This of course leads to the question of how to choose *K*, which we address below. In a sparser network, of course, the complexity will depend on the two nodes chosen as endpoints (those from denser clusters will have larger runtimes), but if the mean degree of the network is ${\mathscr {D}}$, then on average the complexity will be more like ${\mathcal {O}}({\mathscr {D}}^K)$. (The Additional file 1 contains figures showing the runtime for our example metabolic network and simulated networks of various sizes for different values of K).

#### Choice of *K*

**Fig. 4:**
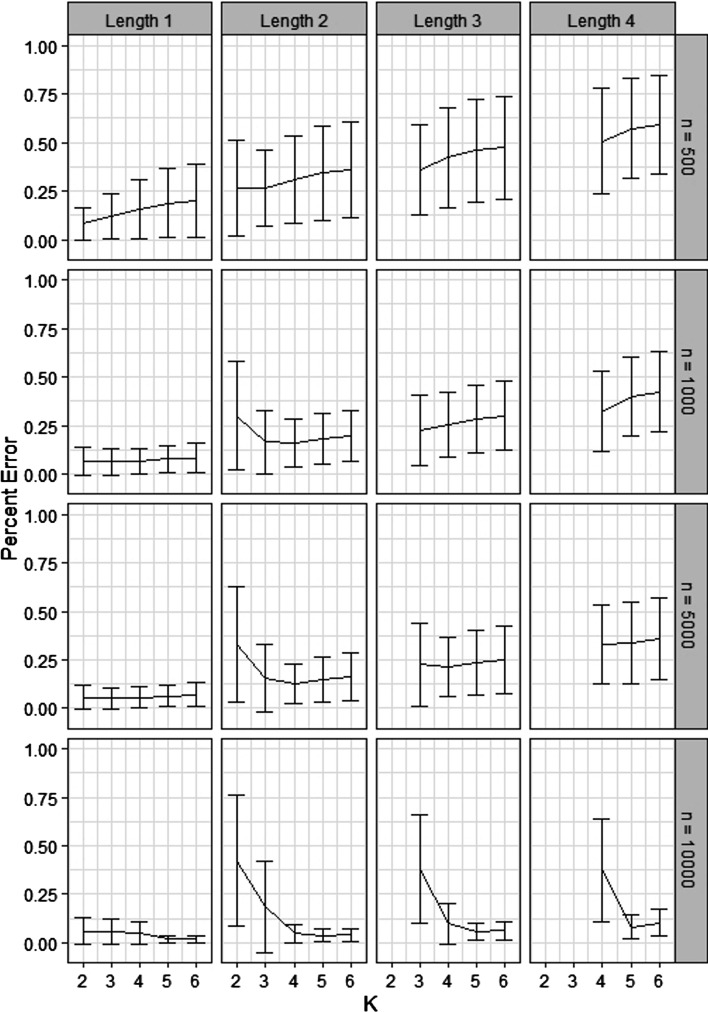
Dependence of PPS estimation accuracy on sample size *n* and *K*. Results are stratified by length of the max PPS path and sample size to aid visualization. Note that in this simulation, $K = 6$ corresponds exactly to ${\hat{s}}_p$ from (13). Error decreases with *n*, but actually increases for a given *n* if *K* is large enough. However, if *n* is not too small (at least 1000, say) the increase in error if *K* is too large is quite small compared to the increase in error if *K* is too small. It is therefore better to err on the side of choosing *K* too large than too small

In order to assess how *K* affects the accuracy of ${\hat{s}}_p^K$, we simulated datasets of varying size ($n = 10^2, 10^3, 10^4$) from a $N(\mathbf{0 }, \Sigma )$, where $\Sigma$ is the variance-covariance structure suggested by the GGM in Fig. 1, using the MASS package [21] in R. In particular, all nodes have a marginal *N*(0, 1) distribution (see the Additional file 1 for the full variance-covariance matrix). The GGM in Fig. 1 was randomly generated, with edges assigned between nodes with probability 0.2, and partial correlations chosen uniformly from $(-1, 1)$. For each dataset, we computed the max PPS $s_{p_{\text {max}}}$ for every pair of nodes in the network using values of *K* ranging from 2 to 6. We excluded the small number of pairs that were connected by paths of length 7 or greater, so that ${\hat{s}}_p^K = {\hat{s}}_p$ exactly. We then compared the estimates with the truth. The results are shown in Fig. 4. Each row represents the results from 100 runs at a fixed sample size. The separate panels within each row stratify the results by $|p_\text {max}|$ (this is for visualization purposes). The *y*-axis in each plot is the percent error, $|{\hat{s}}^K_{p_{\text {max}}} - s_{p_{\text {max}}} | / s_{p_{\text {max}}}$. The mean error over the 100 runs is plotted, and error bars represent one standard deviation.

The results provide a good illustration of the influence of *K* and *n* on estimation of PPS. First, recall that *K* governs how close ${\hat{s}}_p^K$ is to ${\hat{s}}_p$, not $s_p$ itself (i.e. the truth). Therefore, we don’t expect the error to go to zero for fixed *n* as *K* grows - it can only be as small as the sample size allows. However, the error does approach zero as *n* increases.

Next, notice that in each trajectory, there is an optimal value of *K* past which the error increases. This may seem counterintuitive, but recall that computing the denominator in (13) requires estimation of $\gamma _p$ for very long paths, many of which will be much smaller than the precision of ${\hat{\gamma }}_p$ we can expect based on *n* (this is because the $\tau _p$ component of (10) decreases in absolute value as path length increases, particularly when partial correlations are small, as they often are in biological data). In that case, an estimate of 0 is actually better in the sense of MSE than ${\hat{\gamma }}_p$. Since ${\hat{s}}_p^K$ effectively estimates ${\hat{\gamma }}_p = 0$ for $|p| > K$, the accuracy decreases slightly when *K* begins to exceed the length of such paths. See [22] for an analogous effect in the estimation of large covariance matrices, where covariances that are expected to be small are set to zero to exploit the same kind of behavior.

Despite the existence of an optimal *K*, in practice we won’t be able to determine it, since we won’t know the ground truth like we do in the simulation setting. Nevertheless, our observations from the simulated data can guide our choice. Notice that if the sample size is not too small, increasing *K* initially produces a large improvement in accuracy. However, past this point, although performace can drop slightly, the results are not nearly as sensitive to *K*. We therefore recommend choosing *K* large enough that the numerical results are relatively stable. This ensures that we are in this low-sensitivity regime of the trajectory.

Based on these observations, we recommend $K = 4$ or 5 as an all-purpose choice of *K*. For a wide range of realistic sample sizes, this allows for the gain in accuracy achieved by increasing *K* while still reaping the variance-stabilizing (and complexity-reducing) benefits of setting ${\hat{\gamma }}_p = 0$ for $|p| > K$. Crucially, for commonly encountered network topologies like scale-free and Erdos-Renyi, the recommended *K* exceeds the expected path length in many cases. For scale-free networks, where the degree distribution follows the power law

15 $$\begin{aligned} {\mathbb {P}}(d) \propto d^{-\lambda }, d = 0, 1, 2, \ldots , \end{aligned}$$

the network diameter is on the order of $\log \log N$, where *N* is the number of nodes, when $2< \lambda < 3$ [23]. This range for the power law exponent is the most frequently encountered in naturally occuring networks [24]. Thus the expected path length between two nodes will be smaller than 4 or 5 unless the network is very large. For an Erdos-Renyi network, the expected path length is given by

16 $$\begin{aligned} \frac{\log p - \gamma }{\log {\mathscr {D}}} + \frac{1}{2}, \end{aligned}$$

where *p* is the size of the network, $\gamma = 0.577$ is the Euler-Mascheroni constant, and ${\mathscr {D}}$ is the mean degree of the network [24, 25]. For a 100 node Erdos-Renyi network, for example, the mean degree would have to fall below 2.4 for the expected path length to exceed 5.

**Fig. 5:**
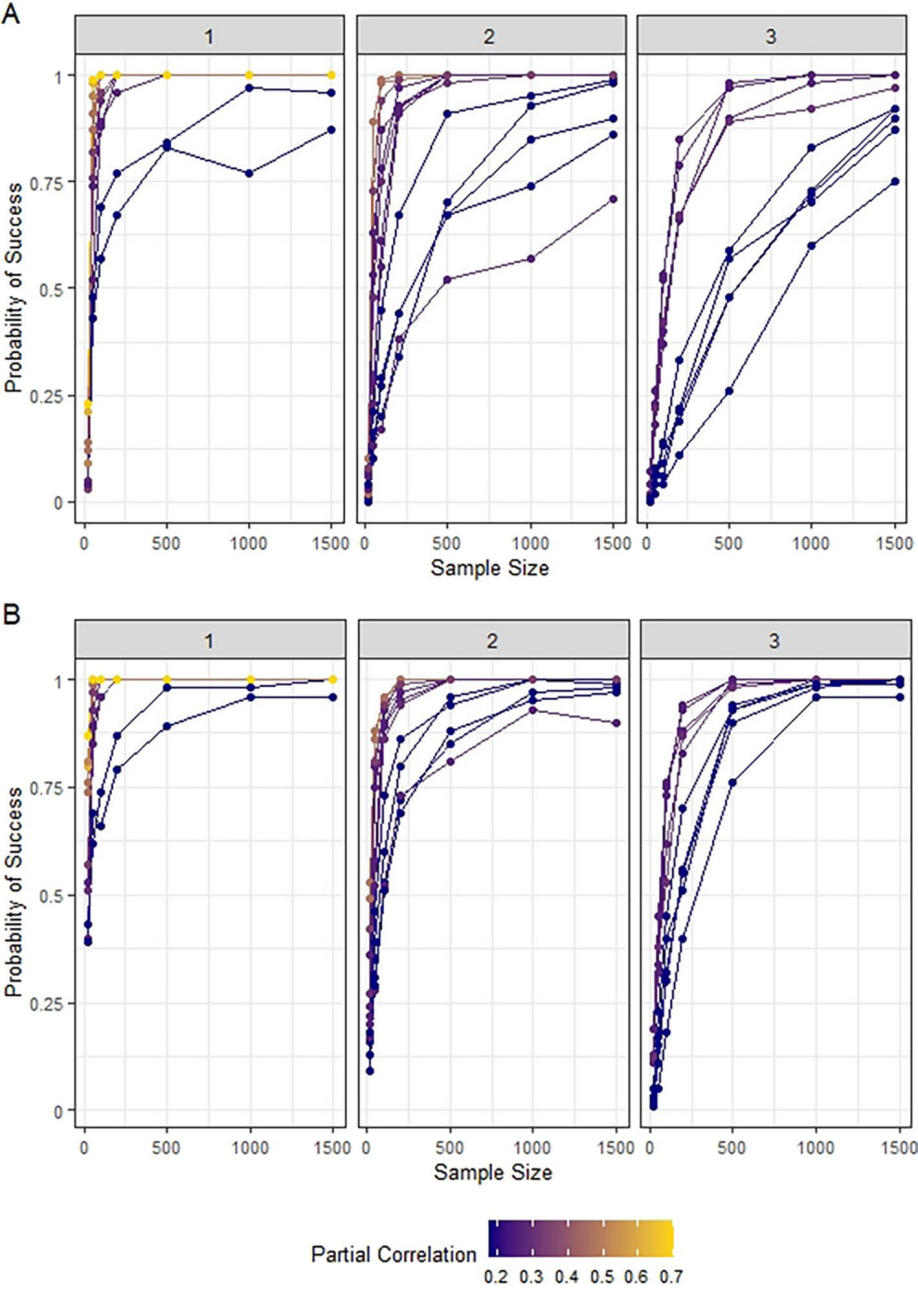
**a** Probability of correct path identification over increasing sample size with no penalization. **b** Probability of correct path over increasing sample size with graphical lasso penalization, with $\lambda$ chosen by cross-validation. In both panels, each line corresponds to a single path, and color represents the magnitude of the smallest partial correlation contained in the path. The subpanels are separated by target path length for readability. These results are with $K = 4$

**Fig. 6:**
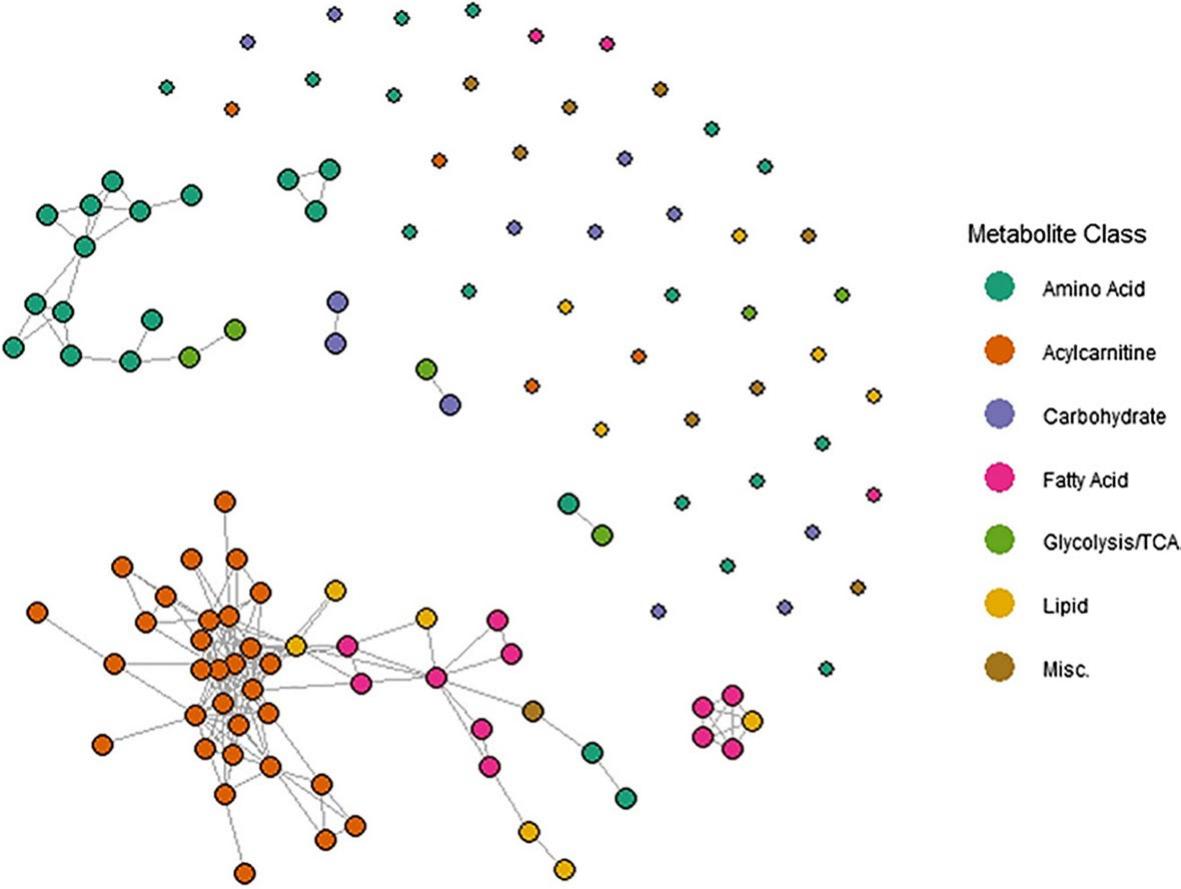
GGM estimated by graphical lasso from the HAPO maternal fasting data. Singletons (degree zero nodes) are depicted smaller

**Fig. 7:**
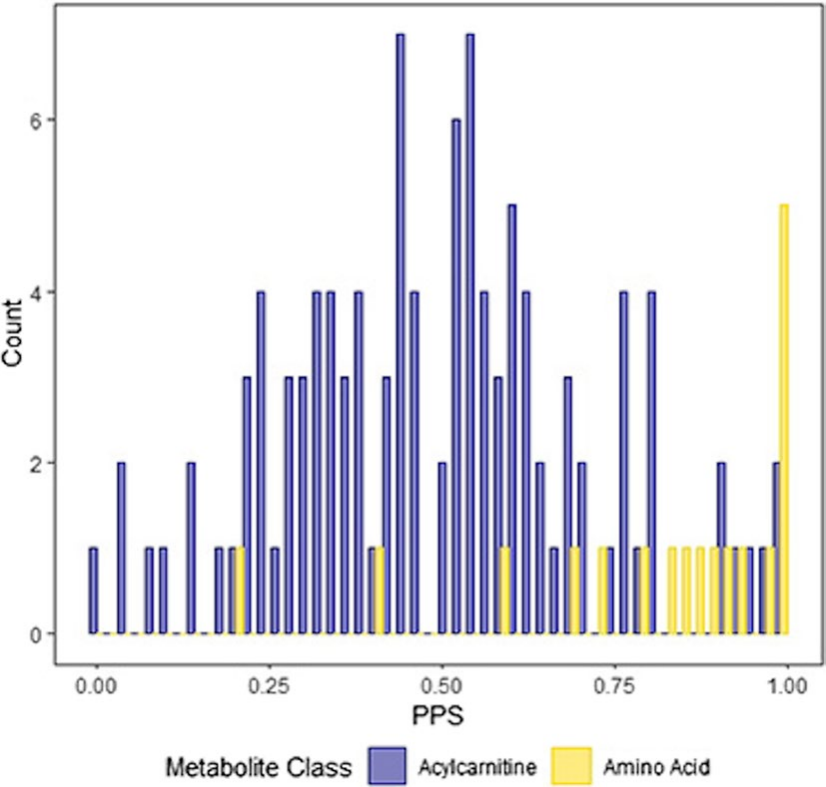
Distributions of PPS for edges connecting pairs of amino acids or acylcarnitines. Edges connecting two amino acids tend to have larger PPS, indicating large relative importance of that edge and the direct connection it signifies (*Note* there were no differences between polar and apolar amino acids). Edges connecting two acylcarnitines have smaller PPS, suggesting less importance of these direct connections compared to the net effect of other network paths

**Fig. 8:**
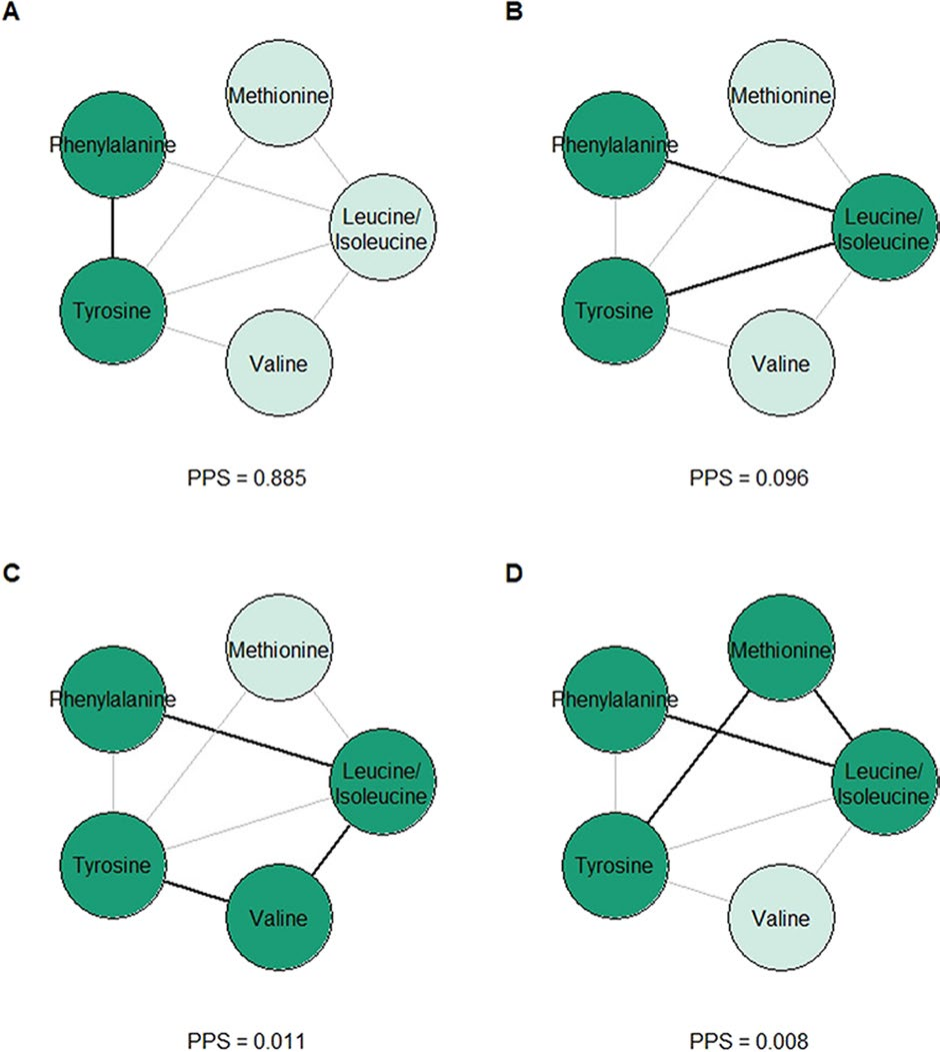
Amino acid subnetwork containing tyrosine and phenylalanine. Conversion (**a**) is the dominant relationship based on PPS

**Fig. 9:**
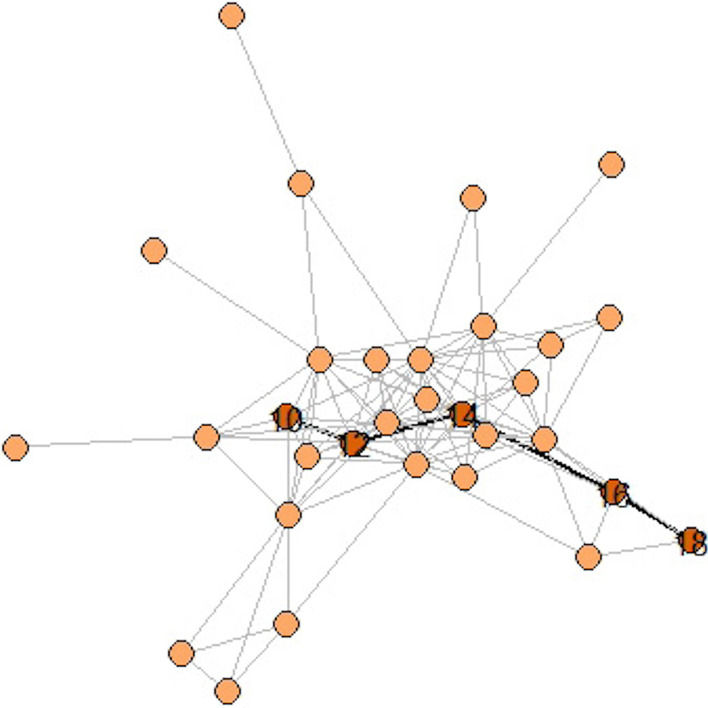
Acylcarnitine subnetwork of the graphical lasso HAPO network. The saturated acylcarnitines C10, C12, C14, C16, and C18 are labeled with carbon numbers, and edges corresponding to addition/removal of two-carbon units are highlighted. These edges comprise the highest PPS paths among these metabolites

**Fig. 10:**
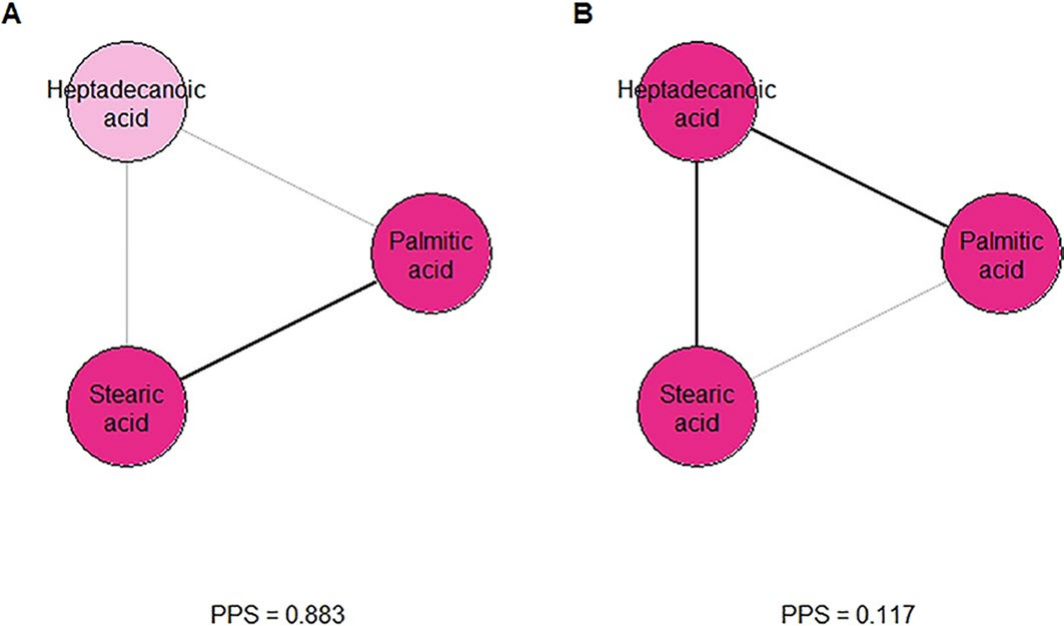
Subnetwork of three saturated fatty acids. The higher PPS path (**b**) corresponds to addition or cleavage of a 2-carbon unit, the more common pathway between stearic and palmitic acid

**Fig. 11:**
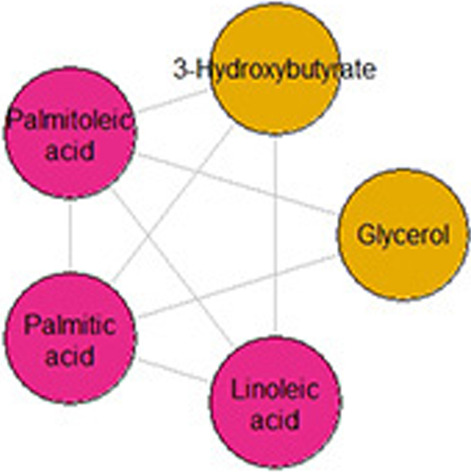
Subnetwork containing palmitic and palmitoleic acid. Despite many possible network paths linking these two fatty acids (including many through metabolites not shown), the edge between them has a high PPS of 0.955. This indicates that the overall network-level relationship between palmitic and palmitoleic acid is dominated by direct conversion

Finally, we point out that the relative ranking of the PPS for two paths does not depend on K, provided both paths have length at most *K*. Notice that the numerator of (14) does not depend on *K*, and that the denominator is the same for all paths. Hence, for two paths $p_1$ and $p_2$ between nodes $i$ and $j$, with $|p_1|,|p_2| \le \kappa$, $|{\hat{s}}_{p_1}| \le |{\hat{s}}_{p_2}|$ if and only if $|{\hat{s}}_{p^K_1}| \le |{\hat{s}}_{p^K_2}|$ for any $K \ge \kappa$. This means that the relative ranking of paths up to length $K$ is the same whether we use $K = \kappa$, $K > \kappa$, or (13). This is useful if we are only interested in identifying the highest PPS paths, rather than precisely estimating the PPS of those paths.

## Results

### Simulation study

PPS estimation will first be demonstrated on the small 10 node GGM shown in Fig. 1c. The small size makes it possible to systematically estimate the PPS of every path between every pair of nodes in the network, and to call attention to the subtleties of PPS estimation. For a variety of sample sizes, we generated 100 datasets from this network using a multivariate normal distribution with mean 0 and covariance matrix chosen so that applying (1) yielded the partial correlation structure depicted in Fig. 1c, which was generated by randomly assigning edges between nodes with probability 0.2 and randomly selecting partial correlations for the edges uniformly from $(-1,1)$. Each node was marginally *N*(0, 1) distributed (see the Additional file 1 for the the full covariance matrix). Data were generated using the MASS package [21] in R. Then, for every pair of nodes in the network, we estimated the PPS of every path between those nodes using (14), $K = 4$, and $\hat{\mathbf{P }}$ equal to the inverse of the empirical Pearson correlation. (The results were similar for $K = 3$ and $K = 5$, see Additional file 1). To judge performance, for each distinct pair of nodes, we compared the true highest PPS path (from our knowledge of the true partial correlation matrix) with the highest estimated PPS path, declaring a success if they were the same, a failure otherwise, for each of the 100 datasets at each sample size. Figure 5a shows the success rates plotted against sample size for this simulation, with subpanels separated by target path length for readability (target path lengths of 4 and 5 are shown in the Additional file 1). Each point represents the number of successes out of 100 for a particular path.

The simulation demonstrates that the ability to identify the highest PPS path between a given pair of nodes depends primarily on three factors: sample size, length of the target path, and the magnitudes of the partial correlations in the target paths. As the sample size increases, accuracy rapidly improves for most paths, but a larger proportion have slower rates of improvement as the path length increases. Because of this, a large sample size ($n > 1000$) is needed to ensure high accuracy for all paths.

Differences in success rate within a given path length is due to the varying sizes of the partial correlations that compose the paths, as indicated by the colors of the trajectories in the figure. Paths composed of larger partials achieve good accuracy at moderate sample sizes. On the other hand, a relatively large sample size is needed for reasonable accuracy in paths with smaller partials. This is a result of the amount of noise in the estimation. Even true zeros (conditionally independent pairs of nodes) will still produce partial correlation estimates with a standard deviation of $\frac{1}{\sqrt{n}}$. For example, if $n = 100$, true partial correlations less than 0.2 are difficult to resolve (18). Longer target paths exacerbate the problem, since their smaller PPS values are harder to differentiate from noisy true zero edge estimates.

We can improve performance in this respect by imposing a sparsity-inducing penalty on the partial correlation estimation. We repeated the simulation using graphical lasso to obtain partial correlation estimates. We again varied the sample size from $n = 20$ to $n = 1500$, and and chose the graphical lasso penalty $\lambda$ using cross-validation. The results, shown in Fig. 5b, demonstrate that large gains in accuracy can be made by reducing noise via penalized estimates.

We note that cross-validation is only one potential method of choosing the penalty parameter $\lambda$. There are many other approaches developed for choosing $\lambda$ in the GGM setting, including EBIC [26], StARS [27], and methods based on false positive rate control [28]. We prefer cross-validation in this setting because it does not depend so much on model assumptions and tends to work well with large sample sizes [27, 29], but this is merely a preference.

An additional benefit of regularization is reduction in computational complexity. The network based on the inverse Pearson correlation is fully connected, making enumeration of all possible paths linking two nodes very expensive. When many partial correlation estimates are exactly zero, we can limit our search to paths not containing those edges, since for such paths ${\hat{s}}_p = 0$.

### HAPO data

Analyses using PPS were also conducted using data from the Hyperglycemia and Adverse Pregnancy Outcome (HAPO) study, an observational, multinational, epidemiological study conducted from 1999 to 2006 to explore associations of maternal glucose levels with adverse pregnancy outcomes [15]. As part of the study, targeted and non-targeted metabolomics assays were performed to measure the concentrations of approximately 130 metabolites present in maternal serum at ~28 weeks gestation (non-targeted assays were performed using gas chromatography–mass spectrometry and results were normalized [30] to account for potential batch variability). Blood samples were obtained from HAPO participants as part of a 75-g oral glucose tolerance test (OGTT). Metabolites were assayed using samples obtained at fasting and 1-hr following consumption of the glucose load. Metabolite profiles from a subset of 1600 study participants, 400 each from four ancestry groups (Afro-Caribbean, Northern European, Mexican American, and Thai) were used for the analysis. All analyses were performed using the full cohort ($n = 1600$). Demographic variables controlled for included study field center, gestational age, maternal height, maternal mean arterial blood pressure, baby sex, number of prior pregnancies, and maternal smoking and drinking status. This dataset has been analyzed in a number of prior studies [13, 30–32].

As with the simulated data, we estimated the GGM underlying the HAPO metabalomics data using both the inverse Pearson correlation matrix and graphical lasso (6). We then applied PPS to selected pairs of metabolites in each estimated GGM to see if the results aligned with known biology. In the sparse network estimated using graphical lasso, we used a maximum path length of 5 edges, i.e. ${\hat{s}}_p^5$ was used to estimate the PPS $s_p$. Since the inverse Pearson correlation network was completely connected, we used a maximum path length of 2 edges for computational feasibility ($K = 3$ already takes 10 min to compute for a single pair of nodes). As explained earlier, while we don’t necessarily expect it to be a good estimate of $s_p$ itself, we can still use ${\hat{s}}^2_p$ as a measure of relative path importance.

### Validation using known biological relationships

In this section, the PPS is validated using known biological relationships among metabolites in the HAPO Study. All networks in this section were constructed using fasting metabolite data.

Amino acids and acylcarnitines are the two most well-represented metabolite classes in the HAPO data and were measured using targeted assays. Acylcarnitines are responsible for transporting long-chain fatty acids across the mitochondrial membrane. Although there are many varieties, they tend to be similar to one another in structure and function, and are often convertible. Amino acids, on the other hand, have distinct structures and functions and are not readily convertible except in specific cases [33, 34].

Based on this biological difference between the two metabolite classes, we might expect the PPS of edges connecting two amino acids to generally be larger than the PPS of edges connecting two acylcarnitines. This is because acylcarnitines tend to have more complex relationships rooted in the combined action of many network paths [33, 34], each of which also contributes to the correlation between their terminal nodes. To see if this was the case, we computed ${\hat{s}}_p^5$ for all edges connecting adjacent pairs of acylcarnitines and adjacent pairs of amino acids in the sparse graphical lasso network (Fig. 6). The edges connecting two amino acids tended to have high PPS (Mean = 0.85, SD = 0.15, taken over all adjacent amino acid pairs), indicating a large relative contribution of that edge to the overall network-level relationship, compared to the edges connecting two acylcarnitines (Mean = 0.47, SD = 0.22, taken over all adjacent acylcarnitine pairs). The histograms in Fig. 7 show the full distribution of these scores for each group separately. Indeed, these results are consistent with the known biology.

The relationship between tyrosine and phenylalanine ($r = 0.440$) is particularly illustrative. The dominant biological relationship between these two amino acids, which differ only by a single hydroxyl group, is direct conversion from phenylalanine to tyrosine. At first glance, the network topology isn’t consistent with this—there is indeed a direct edge between the two amino acids, but there are also many other network paths connecting them. Figure 8 shows the paths connecting tyrosine and phenylalanine and their corresponding PPS in the graphical lasso network. With a much larger PPS, the direct edge dominates the other paths as expected. In the inverse Pearson correlation network, the direct edge remains the highest PPS path between tyrosine and phenylalanine.

The saturated acylcarnitines C10, C12, C14, C16, and C18 provide a good illustration of the structure of the complex acylcarnitine subnetwork (Fig. 9). These metabolites are known to convert by addition and, especially, removal of two-carbon units. Indeed, between C10 and C14 ($r = 0.514$), the highest scoring path in the graphical lasso network is the expected length 2 path C10-C12-C14, with ${\hat{s}}_p = 0.299$ (the next highest path has ${\hat{s}}_p = 0.053$). The results are similar for paths between C12 and C16 ($r = 0.390$), and C14 and C18 ($r = 0.412$). The highest scoring path between C10 and C16 ($r = 0.296$) is the expected length 3 path C10-C12-C14-C16, though between C12 and C18 ($r = 0.308$), C12-C14-C16-C18 is second highest. Figure 9 shows the acylcarnitine subnetwork of the graphical lasso HAPO network, with the edges comprising these paths highlighted. In the fully-connected inverse Pearson correlation network, the highest scoring paths remain the ones we expect. This result in particular demonstrates that while shorter paths do tend to have higher ${\hat{s}}_p$ (since there are fewer factors in $a_p$), it is not always the case that a path of length 1 (an edge) will have a higher ${\hat{s}}_p$ than a path of length 2 or higher.

Fatty acid metabolism is also featured prominently in the HAPO metabolomics data. Consider the subnetwork of the graphical lasso network in Fig. 10. There are two network paths connecting the 18-carbon stearic acid and the 16-carbon palmitic acid ($r = 0.702$). One is the direct edge, and the other passes through heptadecanoic acid (17 carbons). The most common biological mechanism of fatty acid metabolism is $\beta$-oxidation, in which fatty acids are broken down two carbons at a time [33, 34]. Hence, we expect the direct edge connecting the 18-carbon stearic acid to the 16-carbon palmitic acid to carry more weight. The PPS aligns with this expectation: the edge has a PPS of 0.883, while the path through heptadecanoic acid has a PPS of 0.117. This latter path is likely due to common dietary sources of these three fatty acids as well as lipolysis, since direct conversion of stearic or palmitic acid to heptadecanoic acid is not a known metabolic pathway in humans [35].

Figure 11 shows a subnetwork containing palmitic acid and palmitoleic acid ($r = 0.725$), two fatty acids that are directly convertible. We expect the edge connecting them to have a high score, and the many other possible paths to have low score. This is indeed what we observe, with the edge having a PPS of 0.955. Like the others, this example demonstrates that the PPS reflects known biology.

It is important to note that our interpretations of the preceding results in terms of conversions between metabolites is based on a priori knowledge of the metabolic pathways involved. As discussed in the “Methods” section, a high PPS for the edge between two metabolites indicates only that there is a large component of their correlation that cannot be explained by the other metabolites in the network. Further interpretation requires additional assumptions outside of the data itself, and in general the same caveats apply here as for interpretations of coefficients in a multiple regression. For example, a common source for two or more metabolites that is not controlled for before computing the graphical model, such as diet in the fatty acid example above, would be a potential confounder. Correlations could also result from the biochemical factors identified in [3]–in particular, a strong partial correlation between two metabolites does not necessarily mean that they are neighbors in a chemical reaction or biochemical pathway. This means that any paths identified by PPS, or any other correlation-based method, cannot be mapped onto biochemical pathways without additional biological information.

### Hypothesis generation using PPS

The preceding section used known biological relationships to validate the PPS, demonstrating that expected pathways indeed score highly using the technique. This involved a targeted application of the PPS, where certain node pairs of interest were singled out for analysis. However, when working with a new or unfamiliar dataset, an investigator may not always know which node pairs will be of interest. The PPS can still be used in this setting in a systematic way, by being applied to all pairs of nodes in a network. Pairs where the PPS profile has certain characteristics can then be picked out, and can form the basis for new biological hypotheses.

**Table 1:** Metabolite pairs with largest differences in max PPS between 1-hr and fasting states

K	Metabolite A	Metabolite B	Fasting max PPS	1-HR max PPS
*Max PPS in 1-HR vs fasting states*				
5	AC C18:1-OH/C16:1-DC	AC C2	0.175 (0.141, 0.334)	0.727 (0.376, 0.871)
4	AC C18:1-OH/C16:1-DC	AC C2	0.190 (0.153, 0.353)	0.756 (0.406, 0.882)
3	AC C18:1-OH/C16:1-DC	AC C2	0.228 (0.206, 0.530)	0.824 (0.471, 0.917)

Confidence intervals are 95% nonparametric bootstrap confidence intervals

As an example of this type of analysis, PPS was used to investigate differences in network structure between the fasting and 1-hr post glucose metabolite measurements. The HAPO study includes metabolomics data from pregnant mothers in both states, and graphical lasso was used to estimate a network of metabolites for each dataset separately. Then, for each network separately, PPS was applied to every pair of metabolites, and for each pair the path with the highest PPS was selected. Table 1 shows the pair with the largest difference in maximum PPS between the two states. Results are shown for $K = 3,\text { } 4, \text { and } 5$. The pair contains two acylcarnitines, and has a much larger max PPS at 1-hr vs fasting. This could suggest critical involvement of these metabolites for glucose metabolism in the 1-hr vs fasting states.

For a second example, we used PPS to investigate differences in network structure between pregnant mothers and newborn babies. In order to identify metabolites that might play different roles in the two populations, we applied the same procedure as for the fasting and 1-hr datasets, only this time averaged the largest PPS for each metabolite over all the other metabolites it was connected with, in order to get a single measure characterizing network involvement for each metabolite (to make one instance of the procedure concrete, for the maternal and newborn data separately, we computed the max PPS of lactate, say, with all the other metabolites. Then for each dataset we took the average of these values, and examined the difference). Metabolites with a lower average PPS have greater network involvement, in the sense that their marginal correlations with other metabolites are the result of a variety of network paths. On the other hand, metabolites with a higher average PPS have less network involvement, in the sense that fewer network paths contribute to their marginal correlations with other metabolites. The metabolite lactate had the largest difference in average PPS between the maternal and newborn datasets (Table 2). This could indicate a differential role of lactate in the metabolisms of newborn babies versus pregnant mothers.

**Table 2:** Differences in lactate PPS between mothers and babies

Population	Avg lactate max PPS over all other metabolites
*Average max PPS of lactate with other metabolites in mothers and babies*	
Babies	0.275 (0.220, 0.401)
Mothers	0.543 (0.460, 0.695)

Results shown with $K = 4$. The confidence intervals are 95% nonparametric bootstrap confidence intervals

### Software

Our method is implemented in the R package pps, available at https://github.com/nathan-gill/pps. It includes an interactive app to visualize subnetworks and high PPS paths in a user’s dataset. A guide for using the app can be found in the Additional file 1, and the data are available on the Northwestern Medicine DigitalHub at https://doi.org/10.18131/g3-4b37-y728.

## Discussion and conclusions

In this paper, we developed a novel scoring method, PPS, for paths in a GGM that measures the relative importance of those paths to the network-level Pearson correlation between their respective terminal nodes. PPS can be used to probe network structure on a finer scale by investigating which paths in a potentially intricate topology are the most significant. Metabolomics data from the HAPO study was used to demonstrate that PPS analysis is consistent with well-documented biological relationships present in real data. PPS is based on the representation of the correlation between two nodes in a GGM as a sum of terms corresponding to network paths connecting those nodes. Adding PPS to the network analysis toolkit may enable researchers to ask new questions about the relationships among nodes in network data.

A limitation of our method is the relatively large sample size needed for reliable identification of the highest PPS path between two nodes in a network, a result of variability in the individual partial correlation estimates (especially the true zeros) propagating to the estimate of PPS itself. We recommend regularization using graphical lasso as a possible way to eliminate some of this variability. Other strategies could include restricting the path search space to a particular subset of nodes, or taking the top $k$ estimated PPS paths instead of the single top path. Further research is needed to develop more efficient estimators of PPS.

Traditionally, correlation-based methods and partial correlation-based methods like GGMs have been treated as distinct approaches to network construction. While it is true that edges in these networks represent different relationships between their nodes (namely conditional vs unconditional), the findings in this paper shed light on the connection between the two, making explicit how a partial correlation structure gives rise to its corresponding correlation structure. The representation (9) can in fact be used to show that in many cases, GGMs and correlation networks built from the same data can be quite similar. A careful study of this observation will be the subject of future work.

### Supplementary Information

**Additional file 1:** Demonstration file for the PPS RShiny application.

**Additional file 2:** Supplementary Information and Figures.

## Data Availability

The data underlying this article are available in the Northwestern Medicine DigitalHub at https://doi.org/10.18131/g3-4b37-y728. Additionally, our method is implemented in the R package pps, available at https://github.com/nathan-gill/pps. It includes an interactive app to visualize subnetworks and high PPS paths in a user’s dataset. A guide for using the app can be found in the supplemental materials.

## Appendix

In order to derive (9) from the main text, we will use the following formulation of the determinant: For a matrix $\mathbf{M } = \{m_{ij}\}$, the determinant is given by

17 $$\begin{aligned} |\mathbf{M }| = \sum _\sigma \text {sgn}(\sigma )m_{\sigma (1), 1}\cdots m_{\sigma (n), n}, \end{aligned}$$

where the sum is over all possible permutations $\sigma$ of the set $\{1,\ldots ,n\}$. The determinant is thus a sum of $n!$ terms, each of which is a product of $n$ entries of the matrix chosen so that each row and column is represented exactly once. An analogy can help make this formula concrete. Imagine walking across the matrix, proceeding from column to column from left to right. Each time, we must visit a new row that we haven’t visited before (note that since the matrix is square this ensures that every row is visited exactly once). During the walk, we keep a running product of the entries of the matrix we visit. Each such product, multiplied by the sign of the permutation to which it corresponds, is a summand in the determinant formula.

We now consider an arbitrary partial correlation matrix $\mathbf{P } = \{\pi _{ij}\}$. Recall that we can obtain the correlation from the partial correlation using the equation

18 $$\begin{aligned} \mathbf{C } = \mathbf{D }^{-1}\mathbf{A }^{-1}\mathbf{D }^{-1}, \end{aligned}$$

where

$$\begin{aligned} \mathbf{A }_{ij} = {\left\{ \begin{array}{ll} - \pi _{ij} &{} i \ne j \\ \pi _{ij} &{} i = j \end{array}\right. }, \end{aligned}$$

and $\mathbf{D }$ is a diagonal matrix with $d_{ii}$ equal to the square root of the (*i*, *i*) entry of $\mathbf{A }^{-1}$. We will begin by focusing on the structure of $\mathbf{A }^{-1}$. The $(i,j)$ entry of $\mathbf{A }^{-1}$ is given by

19 $$\begin{aligned} (-1)^{(i+j)}\frac{|\mathbf{A }_{(i,j)^*}|}{|\mathbf{A }|}, \end{aligned}$$

where $\mathbf{A }_{(i,j)^*}$ denotes the submatrix of $\mathbf{A }$ with row $i$ and column $j$ removed. Since $\frac{1}{|\mathbf{A }|}$ is common to all entries of $\mathbf{A }^{-1}$, it will eventually be canceled when we normalize to obtain the correlation. Hence, we can ignore it, and focus on the numerator $|\mathbf{A }_{(i,j)^*}|$.

By symmetry, it suffices to consider $|\mathbf{A }_{(1,2)^*}|$, that is,

20 $$\begin{aligned} \begin{vmatrix} a_{12}&a_{23}&\cdots&a_{2n} \\ a_{13}&a_{33}&\cdots&a_{3n} \\ \vdots&\vdots&\ddots&\vdots \\ a_{1n}&a_{3n}&\cdots&a_{nn} \end{vmatrix}. \end{aligned}$$

Consider the individual terms that appear in this determinant (i.e. the individual $(n-1)!$ summands in (17) applied to $\mathbf{A }_{(1,2)^*}$). In every summand in the determinant, the indices $1$ and $2$ will each appear exactly once. This is because the index 1 appears in all entries of column 1, but nowhere else, and similarly, the index 2 appears in every entry of row 1 but nowhere else. Since we choose one and only one entry from column 1, and one and only one entry from row 2, indices 1 and 2 will each appear exactly once (note that their appearances could coincide if we choose $a_{12}$). On the other hand, each of the indices $3, \ldots , n$ appears in every entry of both a row *and* a column. For example, the index 3 appears in every entry of row 2 and every entry of column 2. So either we choose $a_{33}$, or we visit row 2 and column 2 separately, each time picking up the index 3 (and similarly for the others up to $n$).

Based on this insight, we can split each product into two parts. The first is a chain of indices beginning with node 1 and ending with node 2. We begin with $a_{1,k_1}$. If $k_1 \ne 2$, we find the second term in which $k_1$ appears, $a_{k_1, k_2}$. Then if $k_2 \ne 2$, we find $a_{k_2, k_3}$, and so on. The second is the terms containing indices not appearing in the chain. Denoting the chain by $c = \{1,k_1,k_2,\ldots ,k_m, 2\}$, we can rewrite every product $a_{\sigma (1), 1}\cdots a_{\sigma (n), n}$ as

21 $$\begin{aligned} a_{\sigma (1), 1}\cdots a_{\sigma (n), n}&= a_{1,k_1}a_{k_1,k_2}\cdots a_{k_m,2}\Pi _{j \notin c }a_{\sigma (j),j} \end{aligned}$$

22 $$= ( - 1)^{{m + 1}} \pi _{{1,k_{1} }} \pi _{{k_{1} ,k_{2} }} \cdots \pi _{{k_{m} ,2}} \Pi _{{j \notin c}} a_{{\sigma (j),j}} .$$

Let $\sigma _{c^*}$ denote the permutation $\sigma$ restricted to those indices not appearing in chain $c$. Note that $\sigma _{c^*}$ is itself a permutation, since for each $j \notin c$, we also have $\sigma _j \notin c$. Let $c(\sigma )$ denote the chain associated with permutation $\sigma$, and let $K_c = \{\sigma : c(\sigma ) = c\}$. Finally, noting that $\text {sgn}(\sigma _{c^*}) = (-1)^{m}\text {sgn}(\sigma )$, we have by applying (17)

23 $$\begin{aligned} (-1)^{(1+2)}|\mathbf{A }_{(1,2)^*}|&= - \sum _\sigma \text {sgn}(\sigma )a_{\sigma (1), 1}\cdots a_{\sigma (n), n} \end{aligned}$$

24 $$= - \sum\limits_{c} {\sum\limits_{{\sigma \in K_{c} }} {{\text{sgn}}} } (\sigma )( - 1)^{{(m + 1)}} \pi _{{1,k_{1} }} \pi _{{k_{1} ,k_{2} }} \cdots \pi _{{k_{m} ,2}} \Pi _{{j \notin c}} a_{{\sigma (j),j}}$$

25 $$= - \sum\limits_{c} {\sum\limits_{{\sigma \in K_{c} }} {{\text{sgn}}} } (\sigma _{{c^{*} }} )( - 1)^{{2m + 1}} \pi _{{1,k_{1} }} \pi _{{k_{1} ,k_{2} }} \cdots \pi _{{k_{m} ,2}} \Pi _{{j \notin c}} a_{{\sigma _{{c^{*} }} (j),j}}$$

26 $$= \sum\limits_{c} {\pi _{{1,k_{1} }} } \pi _{{k_{1} ,k_{2} }} \cdots \pi _{{k_{m} ,2}} (\sum\limits_{{\sigma \in K_{c} }} {{\text{sgn}}} (\sigma _{{c^{*} }} )\Pi _{{j \notin c}} a_{{\sigma _{{c^{*} }} (j),j}} )$$

27 $$= \sum\limits_{c} {\pi _{{1,k_{1} }} } \pi _{{k_{1} ,k_{2} }} \cdots \pi _{{k_{m} ,2}} |{\mathbf{A}}_{{c^{*} }} |.$$

Some of the chains $c$ between nodes 1 and 2 will contain consecutive nodes whose partial correlation is zero. In fact, if ${\mathscr {P}}_{12}$ is, as in the main text, the set of network paths connecting nodes 1 and 2, then

$$\begin{aligned} {\mathscr {P}}_{12} = \{c : \pi _{1,k_1}\pi _{k_1,k_2}\cdots \pi _{k_m,2} \ne 0\}. \end{aligned}$$

Therefore, letting $\tau$ be as in the main text, and replacing $c$ with $p$ to emphasize that now we are dealing only with the subset of chains corresponding to network paths,

$$\begin{aligned} (-1)^{(1+2)}|\mathbf{A }_{(1,2)^*}| = \sum _{p \in {\mathscr {P}}_{12}}\tau _p|\mathbf{A }_{c^*}|. \end{aligned}$$

Normalizing then gives the desired result.
