# Correction: Path-level interpretation of Gaussian graphical models using the pair-path subscore

**DOI:** 10.1186/s12859-022-04990-7

**Published:** 2022-11-04

**Authors:** Nathan P. Gill, Raji Balasubramanian, James R. Bain, Michael J. Muehlbauer, William L. Lowe, Denise M. Scholtens

**Affiliations:** 1 Feinberg School of Medicine, Northwestern University, Chicago, IL USA; 2 Department of Biostatistics and Epidemiology, University of Massachusetts - Amherst, Amherst, MA USA; 3 Sarah W. Stedman Nutrition and Metabolism Center, Duke University Medical Center, Durham, NC USA; 4 Duke Molecular Physiology Institute, Durham, NC USA; 5 Duke University School of Medicine, Durham, NC USA

## Correction to: BMC Bioinformatics (2022) 23:12 https://doi.org/10.1186/s12859-021-04542-5

Following publication of the original article [1], the authors would like to add additional references and a paragraph under the heading Methods. The additional paragraph and references are given below.

Equation (9) had previously been stated in [2]. This paper goes on to use what we have called γp (equation (10), the unsigned numerator in PPS) as a measure of the contribution of a path to the correlation between its terminal nodes. Additional papers ([3] and [4]) discuss the interpretation of these path weights and expand the concept to path-level decompositions of other measures of association between network nodes. We note that, in these papers, the quantity of interest is γp, whereas in this paper the quantity of interest is the PPS (12), and we provide a detailed account of its properties and behavior when applied to real data. A key difference between the PPS and the γp is that the PPS measures the proportion of the correlation attributable to a path, whereas γp gives the raw contribution. Also distinctive in our paper is the availability of a software package to implement PPS. Our software can also be used to implement the methods of [2], [3], and [4], since the γp themselves are also available.

[2] Jones, B., West, M.: Covariance decomposition in undirected gaussian graphical models. Biometrika 92, 779–786 (2005)

[3] Roverato, A., Castelo, R.: The networked partial correlation and its ap- plication to the analysis of genetic interactions. Journal of the Royal Statistical Society Series C, 647–665 (2016)

[4] Roverato, A., Castelo, R.: Path weights in concentration graphs. Biometrika 107, 705–722 (2020)

The original article [1] has been corrected.

## References

[1] Gill NP (2022). Path-level interpretation of Gaussian graphical models using the pair-path subscore. BMC Bioinform.

