# Supplementary material for: Path-level interpretation of Gaussian graphical models using the pair-path subscore
Source: BMC Bioinformatics. 2022 Jan 5;23:12. doi: 10.1186/s12859-021-04542-5 (PMC8729005; doi:10.1186/s12859-021-04542-5)
Supplement: Supplementary file 1 — Additional file 1: Demonstration file for the PPS RShiny application. [file 12859_2021_4542_MOESM1_ESM.pdf]

PPS App Demo

The R package `pps` includes an interactive RShiny application for exploration and vizualization of PPS analyses. Broadly speaking, the app builds a Gaussian Graphical Model (GGM) from a user's data, and then applies PPS to that GGM. This document provides an introduction to the app via a HAPO data example, available in the Northwestern Medicine DigitalHub at https://doi.org/10.18131/g3-4b37-y728.

Installing the package

The package can be installed and loaded using the following commands.

```
library(devtools)

devtools::install_github("nathan-gill/pps")

library(pps)
```

Running the app

After loading the `pps` package, the app can be run by calling the function `run.PPS.app()`. The app will launch in a new window, and the homescreen is shown in Figure 1.

Figure 1: Homescreen for the PPS app.

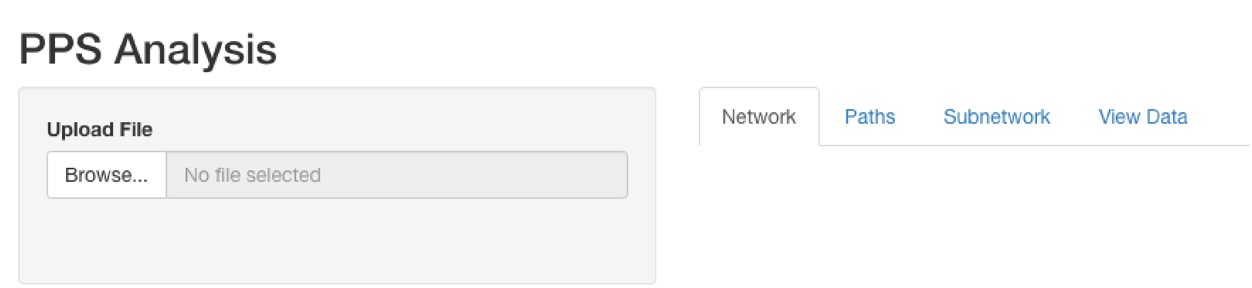

Load data

Click the "Browse" button to open your computer's file explorer. From here, navigate to your desired .csv or .xlsx file. This file should contain an $n \times p$ numeric data matrix that is ready for input into the graphical lasso algorithm. The file should have column headings corresponding to the names of the nodes - these will be used as labels in the results.

Select GGM and PPS parameters

Once a dataset is loaded, input fields for the model parameters will appear. The first is for the graphical lasso penalty parameter, i.e. λ in the precision matrix estimator estimator

$$\hat{\Omega} = \text{argmin}_{\Omega}\left(\text{tr}(S\Omega) - \log\det\Omega + \lambda\sum_{j\neq k}|\Omega_{jk}|\right),$$

where S is the empirical covariance matrix of the data. For any chosen λ, the estimated GGM will appear onscreen, as shown in Figure 2. The column headings from the data will be used to label the nodes.

Figure 2: GGM vizualized after selecting λ and terminal nodes.

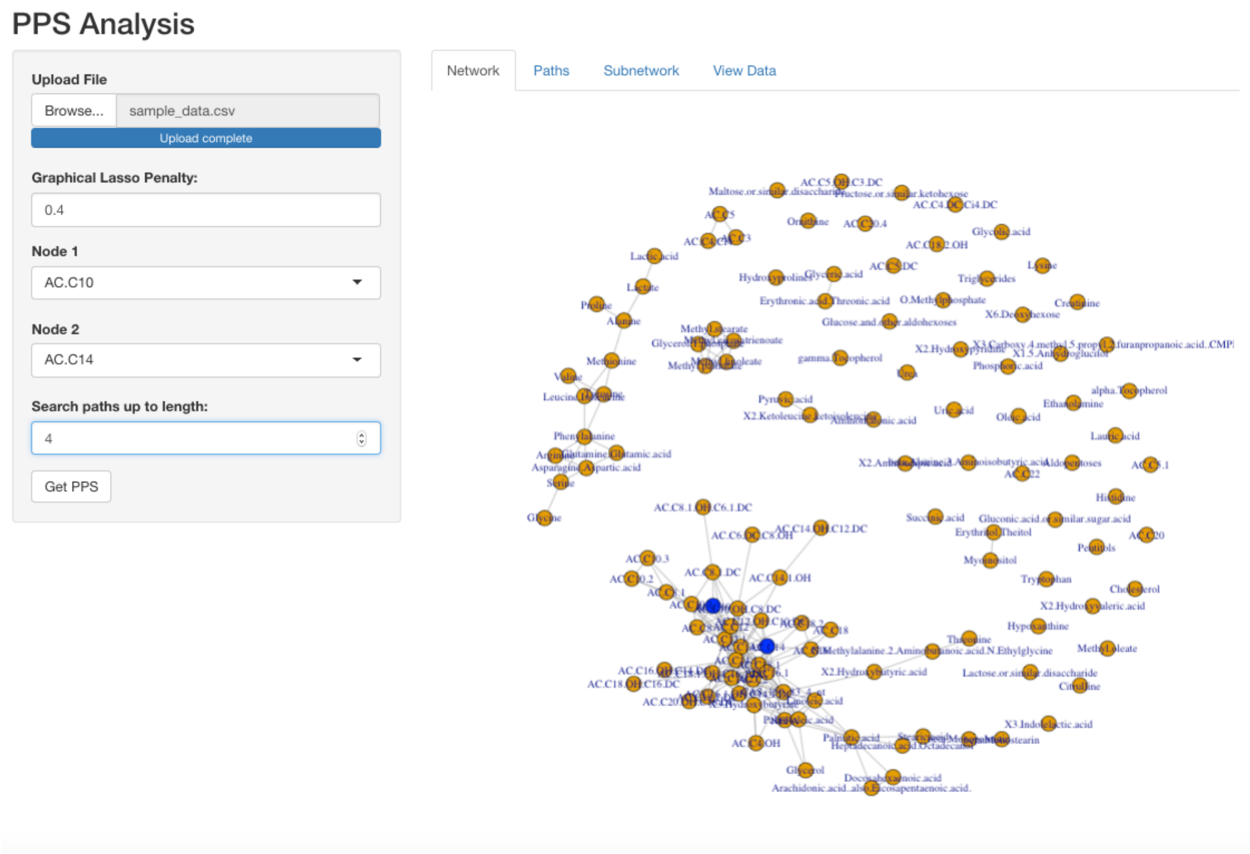

Next, select the two terminal nodes to which you would like to apply PPS. The drop-down menus under "Node 1" and "Node 2" will contain the user-supplied node names. The selected nodes will appear in blue in the network (Figure 2).

Finally, select the maximum path length (K in the paper) up to which to search for network paths between the chosen nodes.

Note: there is nothing special about the default values for these parameters, and they should not be viewed as suggestions. The max path length should not exceed 5 unless the network is small (smaller p) or sparse (larger λ), since computation time can become intensive rather quickly.

Check that the data file was read properly

If something unexpected has happened thus far, it is a good idea to check that the data file was read properly. The "View Data" (Fig 3) tab prints the $n \times p$ data matrix back to the user so that issues can be diagnosed. The column headings should be node names, and the entries should be individual observations of that node.

Figure 3: The "View Data" tab allows user to check that their data was read properly by the app.

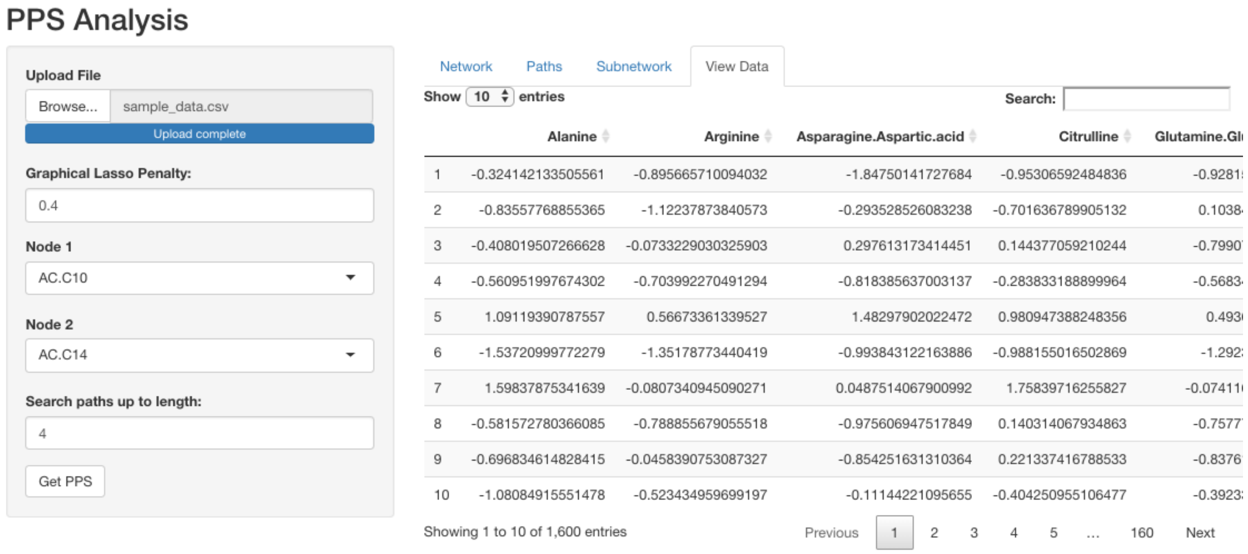

Run PPS

Once all the parameters have been set, pressing the “Get PPS” button will apply PPS to the graphical model currently appearing onscreen. The “Paths” tab contains a list all paths of length at most K connecting the two selected terminal nodes, along with the respective PPS values, in order of decreasing PPS (Fig 4).

The “Subnetwork” tab shows the union of the top 20 PPS paths between the selected terminal nodes (Figure 5). The terminal nodes are again shown in blue, and the edges comprising the highest PPS path are bolded.

Figure 4: The "Paths" tab lists all paths connecting the selected terminal nodes in order of decreasing PPS.

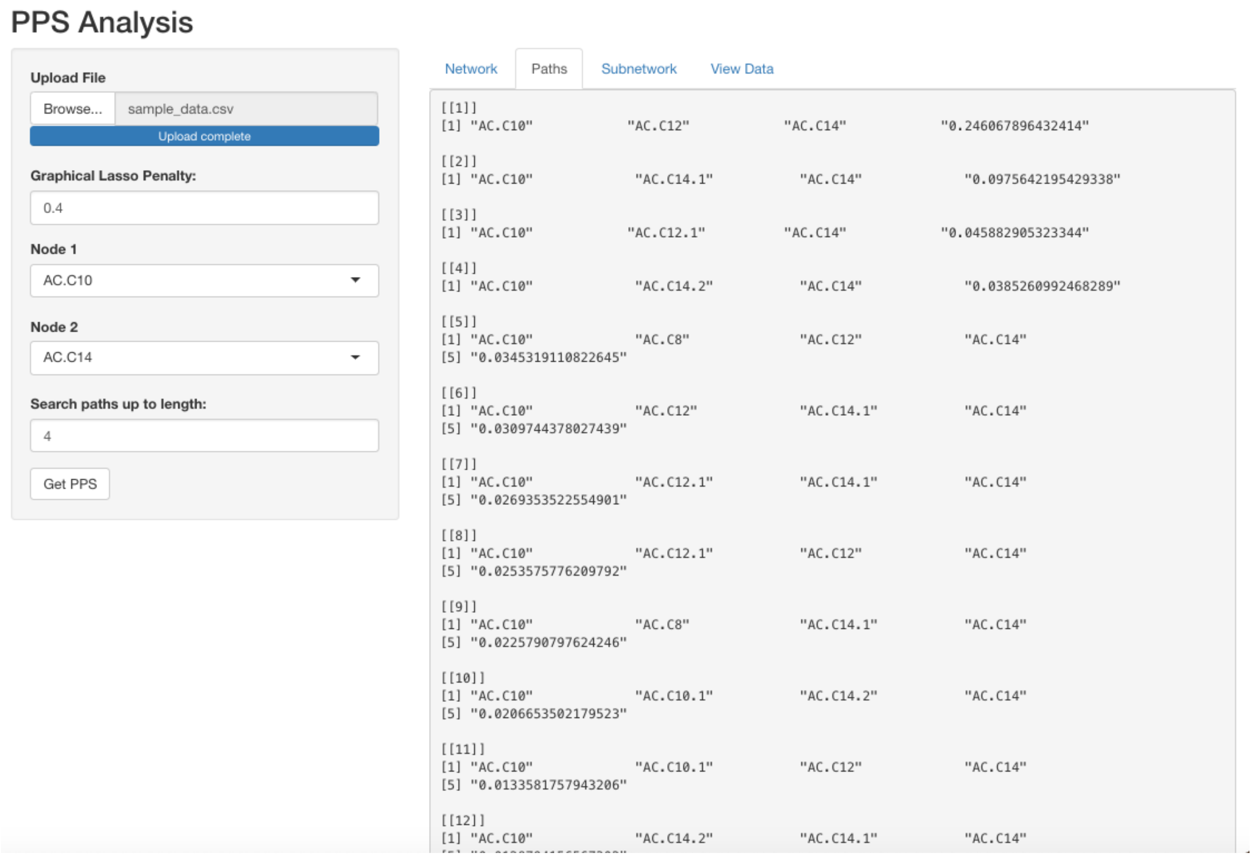

Figure 5: The "Subnetwork" tab shows the union of the top 20 PPS paths, with the terminal nodes in blue and highest PPS path bolded.

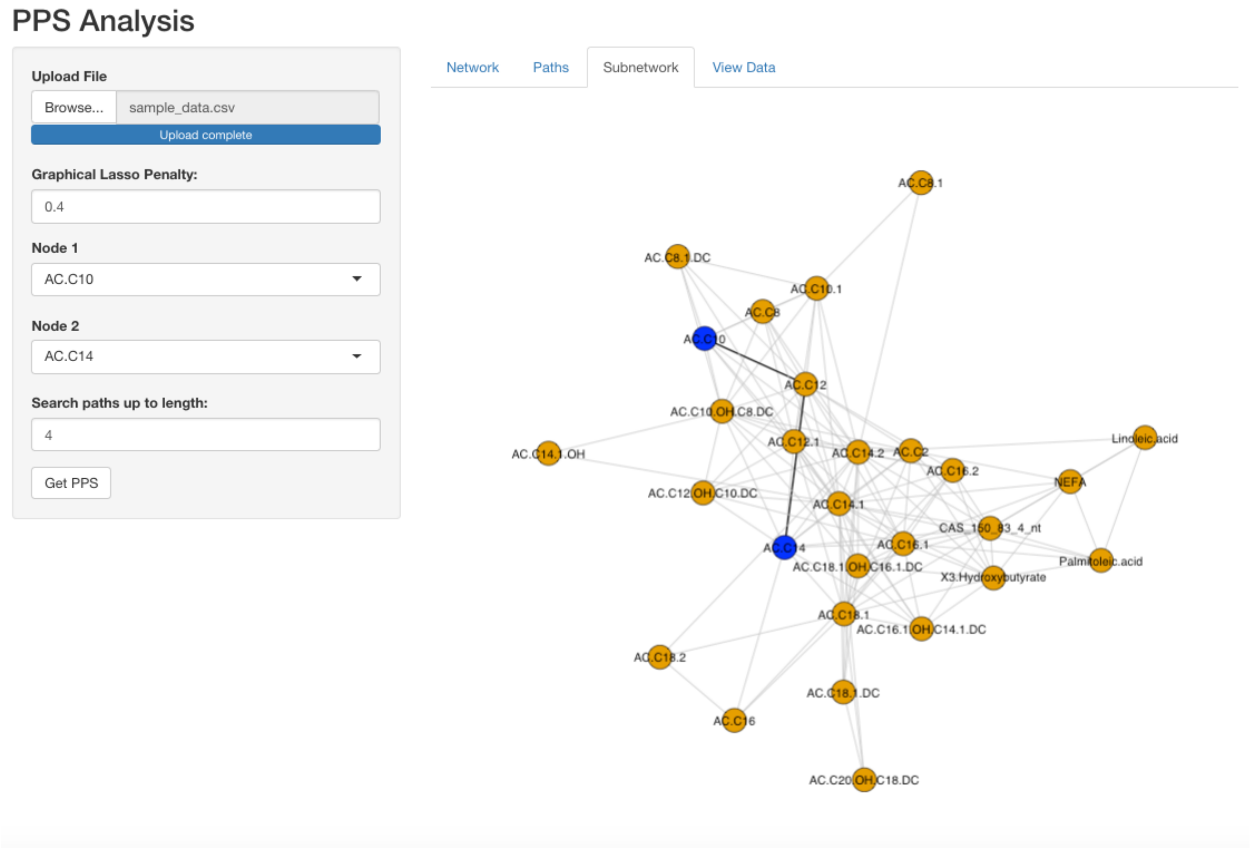

PPS Analysis Outside the App

We can perform the same analysis without the app in the R console. The main function is

```
pps(P, i, j, K = 5, prec = TRUE, use.names = TRUE).
```

Here, `P` is a precision matrix or partial correlation matrix, `i` and `j` are the nodes (i.e. row numbers in `P`) to use as endpoints. If the matrix has named columns, these names can be used for `i` and `j`. `K` is the maximum path length, `prec` indicates whether `P` is a precision matrix or partial correlation matrix, and `use.names` indicates whether the results should be shown in terms of node numbers of node names. The output is a list with the following elements.

`path` A list of paths between nodes i and j in order of descending PPS.

`pps` A list of the PPS values for the paths in path.

`gamma` The individual contributions of each path in path on the correlation scale.

Note that this information can also be found by typing `?pps` into the console.

Example

The analysis shown above in the app can be performed in the console using the following commands, if the data matrix is stored in a matrix called `data`.

```
res <- glasso::glasso(data, lambda = 0.4) # Estimate precision matrix with graphical
lasso

prec <- res$wi # Extract the precision matrix

pps_res <- pps(P = prec, i = "AC C10", j = "AC C14", K = 4, prec = TRUE, use.names =
TRUE) # Run PPS for nodes of interest

pps_res$path[1:20] # Top 20 PPS paths

pps_res$pps[1:20] # PPS of those paths
```
