# Supplementary material for: Path-level interpretation of Gaussian graphical models using the pair-path subscore
Source: BMC Bioinformatics. 2022 Jan 5;23:12. doi: 10.1186/s12859-021-04542-5 (PMC8729005; doi:10.1186/s12859-021-04542-5)
Supplement: Supplementary file 2 — Additional file 2: Supplementary Information and Figures. [file 12859_2021_4542_MOESM2_ESM.pdf]

Supplemental Material

Simulation Results for Other Values of K

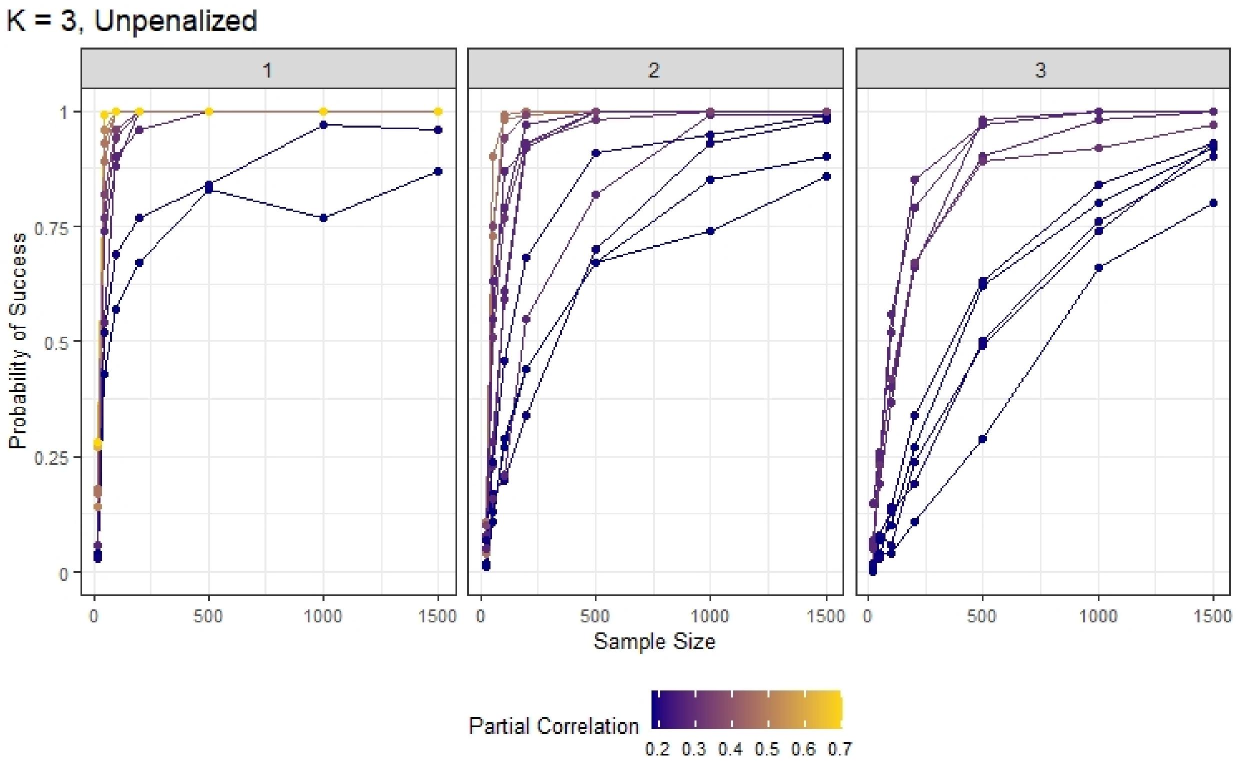

Figure 1: Probability of correct path identification over increasing sample size with no penalization. Each line corresponds to a single path, and color represents the magnitude of the smallest partial correlation contained in the path. The subpanels are separated by target path length for readability.

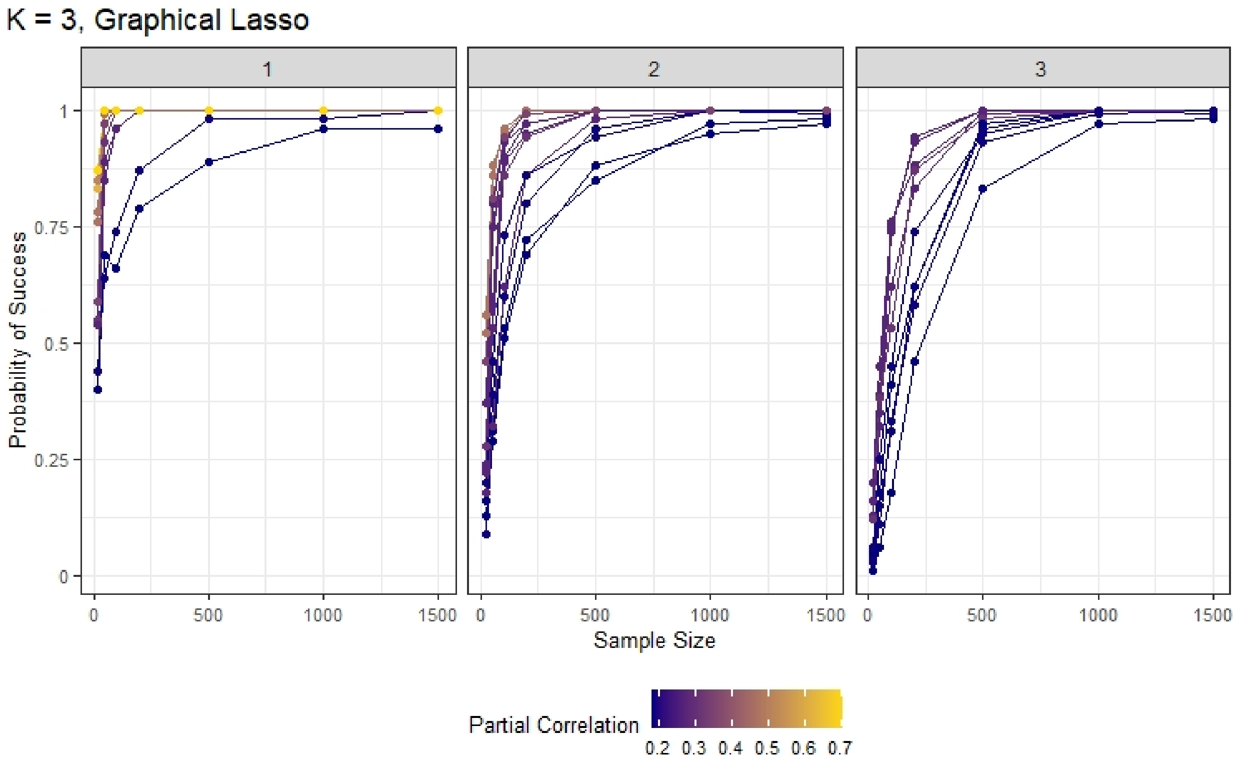

Figure 2: Probability of correct path identification over increasing sample size with graphical lasso penalization, with λ chosen by cross-validation. Each line corresponds to a single path, and color represents the magnitude of the smallest partial correlation contained in the path. The subpanels are separated by target path length for readability.

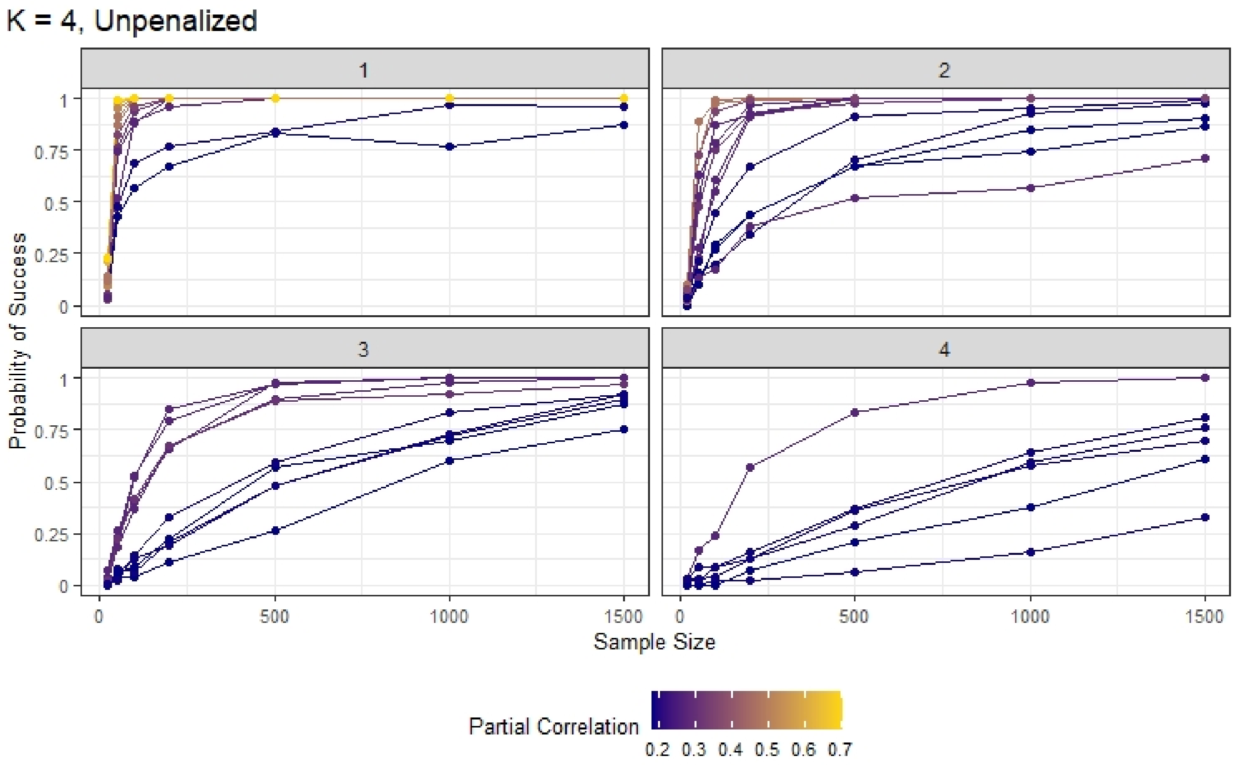

Figure 3: Probability of correct path identification over increasing sample size with no penalization. Each line corresponds to a single path, and color represents the magnitude of the smallest partial correlation contained in the path. The subpanels are separated by target path length for readability.

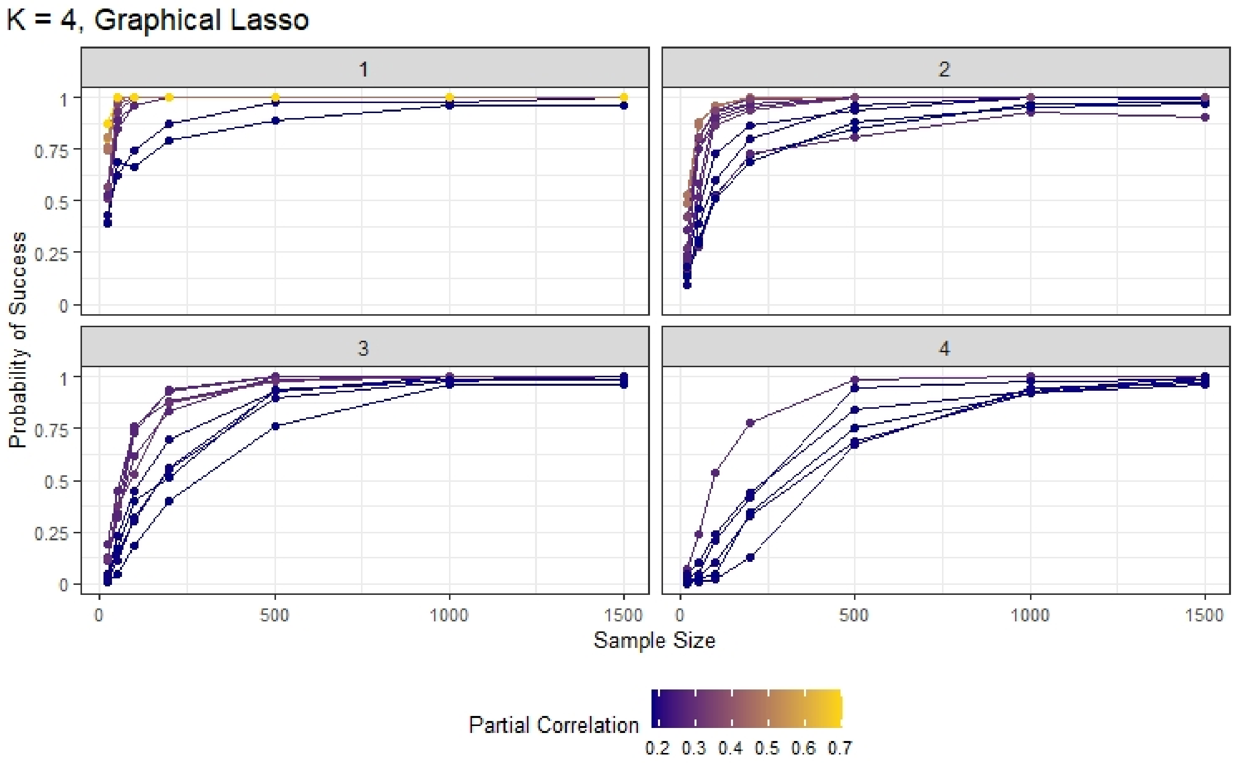

Figure 4: Probability of correct path identification over increasing sample size with graphical lasso penalization, with λ chosen by cross-validation. Each line corresponds to a single path, and color represents the magnitude of the smallest partial correlation contained in the path. The subpanels are separated by target path length for readability.

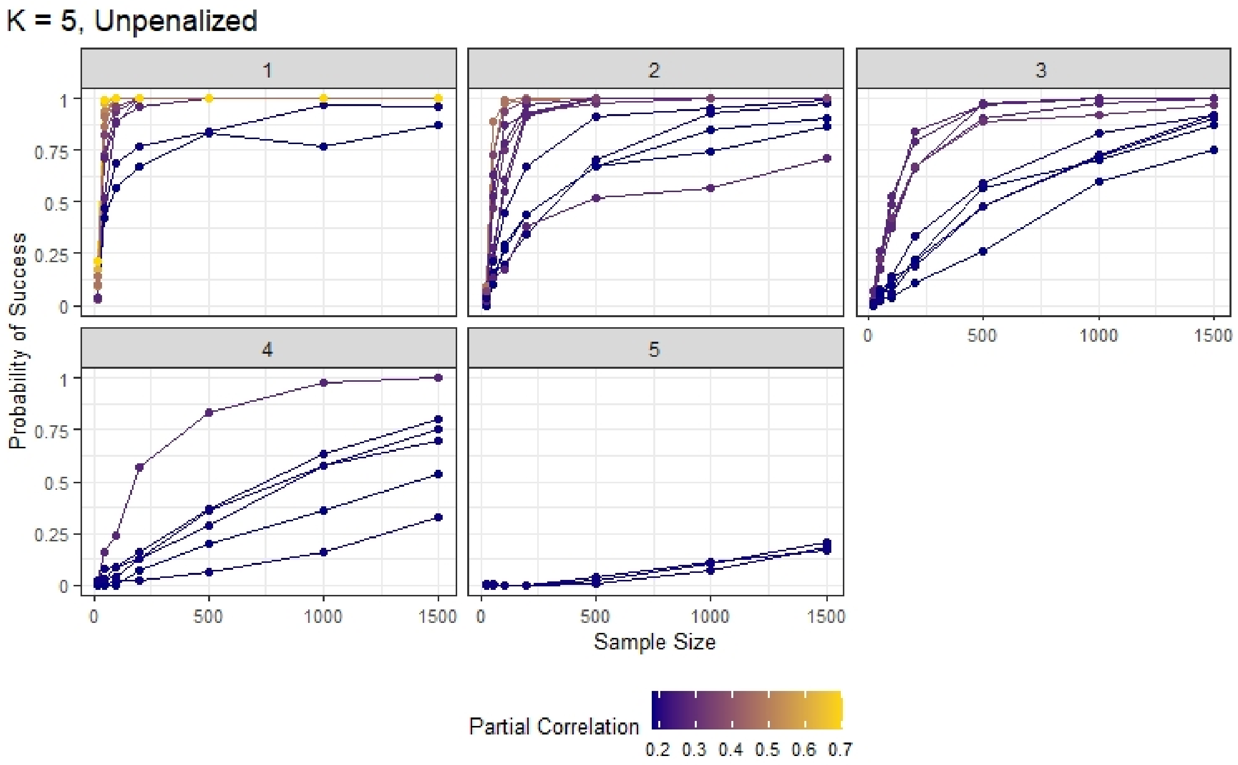

Figure 5: Probability of correct path identification over increasing sample size with no penalization. Each line corresponds to a single path, and color represents the magnitude of the smallest partial correlation contained in the path. The subpanels are separated by target path length for readability.

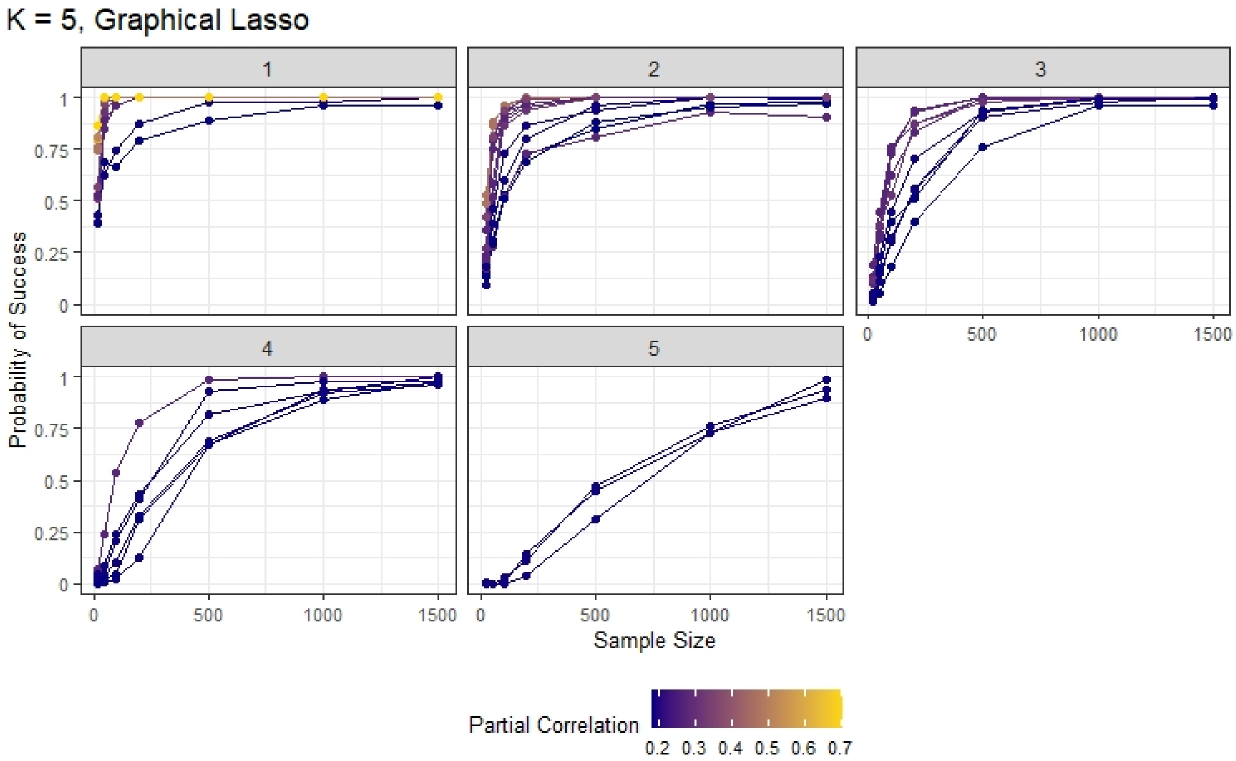

Figure 6: Probability of correct path identification over increasing sample size with graphical lasso penalization, with λ chosen by cross-validation. Each line corresponds to a single path, and color represents the magnitude of the smallest partial correlation contained in the path. The subpanels are separated by target path length for readability.

Runtime

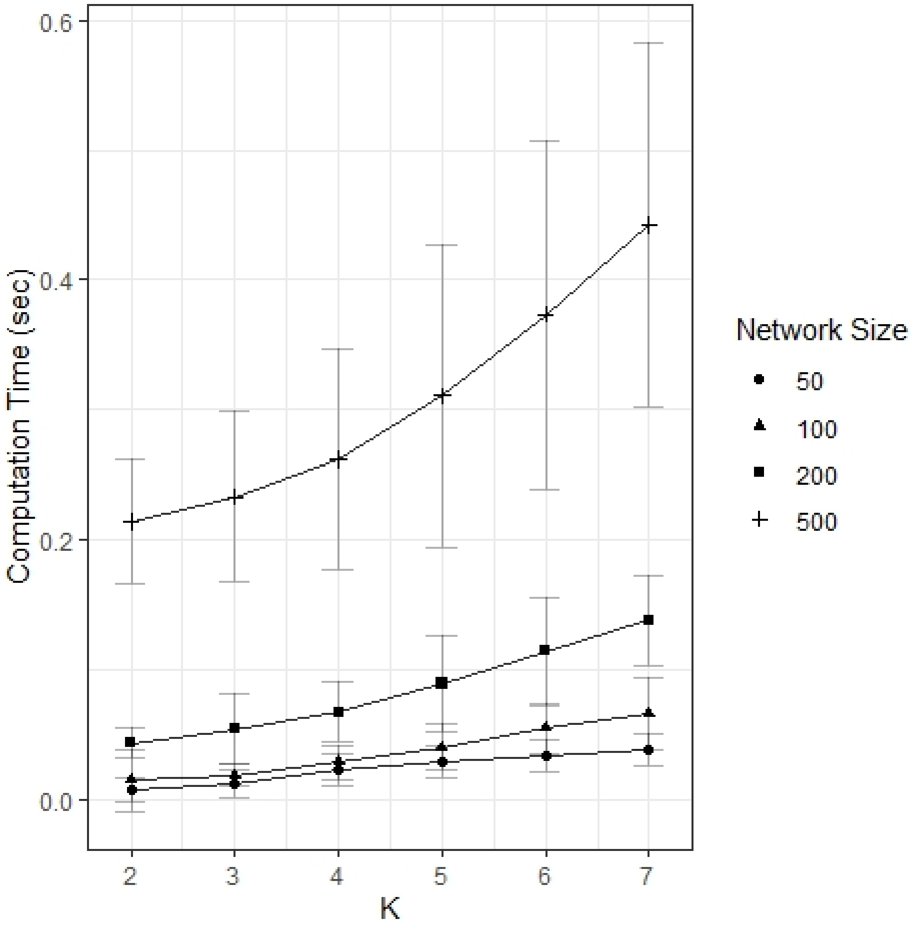

Figure 7: Runtime for various network sizes p and various values of K. Each plotted point corresponds to a distinct pairing of network size and K. For each p, a scale free network of size p was generated, and 20 nodes were selected at random. For all 190 pairings of these nodes, $\hat{s_p^K}$ was used to estimate the PPS of all paths connecting that pair, and the runtime on a Lenovo ThinkPad laptop was recorded. The plotted points represent the average runtime over the 190 pairs, and error bars represent one standard deviation.

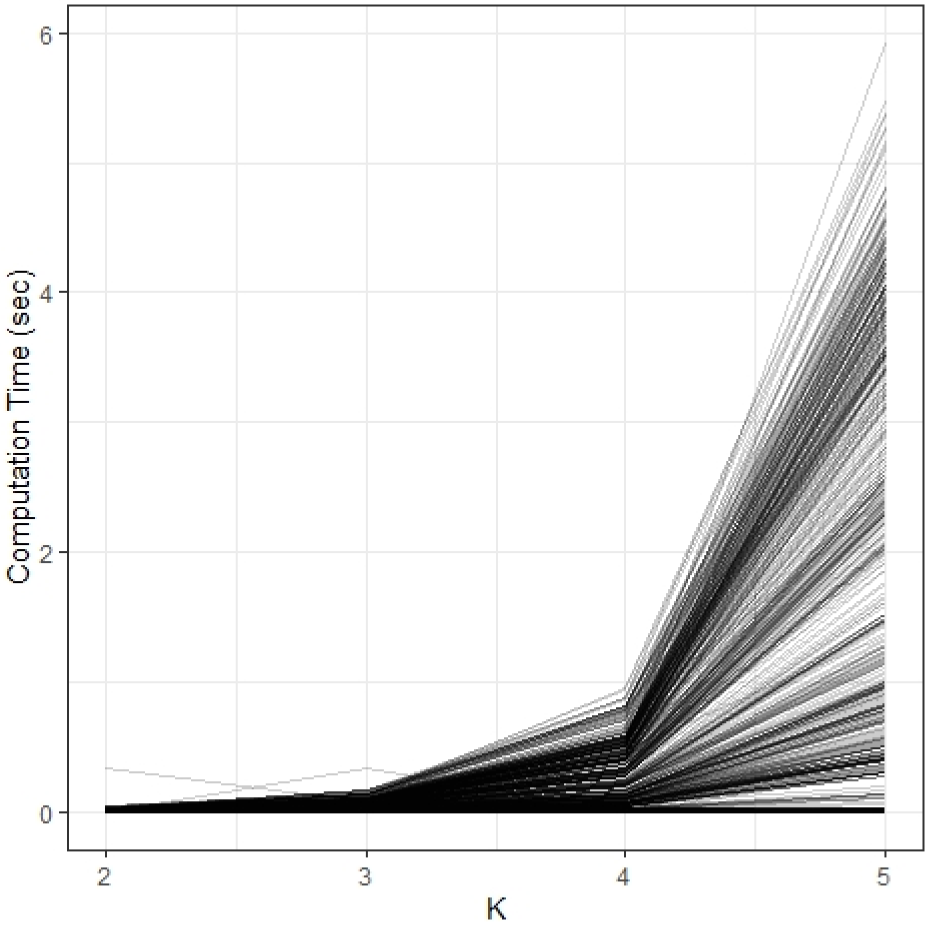

Figure 8: Spaghetti plot of runtime for all pairs of nodes in the acylcarnitine subnetwork (Figure 9 in the main text). Each trajectory corresponds to computing the PPS of every path connecting a single pair at a particular value of K. Note that the runtime is exponential in K, but that there is wide variability in the absolute runtime depending on how many paths connect the two nodes. There are also a few pairs that appear not to increase in runtime with K. These are the nodes on the periphery of the network that are only connected by a handful of paths. Increasing K does not have an effect here because there are so few paths to begin with.

Partial Correlation and Covariance Matrices Used in Simulation

Partial Correlation

Note that this is the partial correlation structure implied by Figure 1(C).

$$\begin{pmatrix} 1 & 0.306 & 0 & 0 & 0.513 & 0 & 0 & 0.178 & 0 & 0 \\ 0.306 & 1 & -0.029 & 0 & 0 & 0 & -0.27 & 0 & 0 & 0 \\ 0 & -0.029 & 1 & -0.618 & 0 & 0 & 0.471 & 0 & -0.267 & 0 \\ 0 & 0 & -0.618 & 1 & 0 & 0 & 0 & 0 & 0 & 0 \\ 0.513 & 0 & 0 & 0 & 1 & 0 & 0 & -0.182 & -0.467 & 0 \\ 0 & 0 & 0 & 0 & 0 & 1 & 0 & -0.346 & 0 & -0.702 \\ 0 & -0.27 & 0.471 & 0 & 0 & 0 & 1 & 0 & 0 & 0 \\ 0.178 & 0 & 0 & 0 & -0.182 & -0.346 & 0 & 1 & 0 & 0 \\ 0 & 0 & -0.267 & 0 & -0.467 & 0 & 0 & 0 & 1 & 0 \\ 0 & 0 & 0 & 0 & 0 & -0.702 & 0 & 0 & 0 & 1 \end{pmatrix} \quad (1)$$

Covariance

$$\begin{pmatrix} 1 & -0.407 & -0.171 & -0.129 & -0.707 & -0.262 & 0.016 & -0.479 & -0.445 & -0.2 \\ -0.407 & 1 & -0.097 & -0.073 & 0.257 & 0.102 & 0.286 & 0.186 & 0.107 & 0.077 \\ -0.171 & -0.097 & 1 & 0.756 & 0.301 & 0.075 & -0.656 & 0.136 & 0.507 & 0.057 \\ -0.129 & -0.073 & 0.756 & 1 & 0.227 & 0.056 & -0.496 & 0.103 & 0.383 & 0.043 \\ -0.707 & 0.257 & 0.301 & 0.227 & 1 & 0.268 & -0.133 & 0.49 & 0.65 & 0.204 \\ -0.262 & 0.102 & 0.075 & 0.056 & 0.268 & 1 & -0.024 & 0.547 & 0.172 & 0.762 \\ 0.016 & 0.286 & -0.656 & -0.496 & -0.133 & -0.024 & 1 & -0.045 & -0.297 & -0.019 \\ -0.479 & 0.186 & 0.136 & 0.103 & 0.49 & 0.547 & -0.045 & 1 & 0.314 & 0.417 \\ -0.445 & 0.107 & 0.507 & 0.383 & 0.65 & 0.172 & -0.297 & 0.314 & 1 & 0.131 \\ -0.2 & 0.077 & 0.057 & 0.043 & 0.204 & 0.762 & -0.019 & 0.417 & 0.131 & 1 \end{pmatrix} \quad (2)$$

3 Node Partial Correlation Matrix in Terms of Correlations

$$\begin{pmatrix} 1 & \frac{r_{12}-r_{13}r_{23}}{\sqrt{(1-r_{23}^2)(1-r_{13}^2)}} & \frac{r_{13}-r_{12}r_{23}}{\sqrt{(1-r_{23}^2)(1-r_{12}^2)}} \\ \frac{r_{12}-r_{13}r_{23}}{\sqrt{(1-r_{23}^2)(1-r_{13}^2)}} & 1 & \frac{r_{23}-r_{12}r_{13}}{\sqrt{(1-r_{13}^2)(1-r_{12}^2)}} \\ \frac{r_{13}-r_{12}r_{23}}{\sqrt{(1-r_{23}^2)(1-r_{12}^2)}} & \frac{r_{23}-r_{12}r_{13}}{\sqrt{(1-r_{13}^2)(1-r_{12}^2)}} & 1 \end{pmatrix} \quad (3)$$
